# Synthesis and Spectroscopic Evaluation of Two Novel Glycosylated Zinc(II)-Phthalocyanines

**DOI:** 10.3390/molecules201018367

**Published:** 2015-10-09

**Authors:** Felix Bächle, Michael Hanack, Thomas Ziegler

**Affiliations:** Institute of Organic Chemistry, Universität Tübingen, Auf der Morgestelle 18, Tübingen 72076, Germany; E-Mails: felix.baechle@uni-tuebingen.de (F.B.); hanack@uni-tuebingen.de (M.H.)

**Keywords:** glycoconjugated phthalocyanine, MEM, CuAAC

## Abstract

In continuation of our work on glycoconjugated phthalocyanines, two new water soluble, non-ionic zinc(II) phthalocyanines have been prepared and fully characterized by means of ^1^H-NMR, ^13^C-NMR, MALDI-TOF, ESI-TOF, UV-Vis spectroscopy, emission spectroscopy and fluorescence lifetime measurements. The carbohydrate-containing phthalonitrile precursors were synthesized through a copper-catalyzed azide-alkyne cycloaddition (CuAAC). The 2-methoxyethoxymethyl protecting group (MEM) was used to protect the carbohydrate moieties. It resisted the harsh basic cyclotetramerization conditions and could be easily cleaved under mild acidic conditions. The glycoconjugated zinc(II) phthalocyanines described here have molar extinction coefficents ε_max_ > 10^5^ m^−1^ cm^−1^ and absorption maxima λ > 680 nm, which make them attractive photosensitizers for photo-dynamic therapy.

## 1. Introduction

Tetrapyrrolic macrocycles are important and ubiquitous natural products. Porphyrins such as heme or chlorophyll are only two examples for the vital role tetrapyrrolic macrocycles play in biological systems. The unique physical, chemical and spectroscopic properties of tetrapyrrole macrocycles account for their rifeness in nature and also provoked great interest in their distinct chemistry and their application as compounds with specific physical properties in material sciences. Although phthalocyanines do not occur in nature these synthetic tetrapyrrolic compounds have surpassed porphyrins with respect to application in material science due to their higher stability, improved spectroscopic properties and their easier accessibility through chemical synthesis compared to porhyrins [1]. For instance phthalocyanines found notable applications in photodynamic therapy (PDT) [2,3,4,5,6,7]. PDT is a modern, non-invasive treatment option for different forms of cancer [8] such as skin, lung, bladder or ophthalmologic cancer forms [9] and is based on the ability of certain dyes to act as photosensitizers (PS) producing molecular oxygen upon irradiation with light. Specifically, in PDT, the photosensitizer is first applied to or made to accumulate in the malign tissue. Upon irradiation with light of a specific wavelength, the accumulated PS is brought to an excited state and generates reactive oxygen species such as singlet oxygen (^1^O_2_) [10] or hydroxyl radicals [11] from oxygen present in the tissue via energy transfer. Singlet oxygen for being a potent oxidant reacts with numerous functional groups of biomolecules in the malign cells leading to apoptosis or necrosis [10].

Due to their outstanding light absorbing capability (high molar extinction coefficients) in the long-wavelength region of the light spectrum a large number of porphyrin type photosensitizers have been developed for use in PDT during the last twenty years. Haematoporphyrin derivatives such as Photofrin [12], Visudyne [13] or Foscan [14] had been among the first commercially available examples of such porphyrin type PS, known as “first generation PS”, and quickly became useful tools for defeating cancer [10]. However, these “first generation PS” typically show only low fluorescence and result in low 1O2 quantum yields. Furthermore, they exhibit a low selectivity towards specific malign tissues and have an absorption maximum (Q-Band) around 630 nm. Light at such wavelengths, however, can only penetrate human tissue to a depth of approximately 5 mm [15]. Additionally, these “first generation PS” were often complex mixtures which made their chemical synthesis and the evaluation of their distinct biological activity difficult [10]. Therefore, the “second generation PS” should overcome these disadvantages, by being chemically pure compounds displaying high solubility in water and having absorption maxima in the range of 670–800 nm which, in turn, allows the light to penetrate human tissue up to 2 cm under optimal circumstances [15]. Likewise, “second generation PS” should display high fluorescence and ^1^O_2_ quantum yields, molar extinction coefficients ε_max_ > 10^5^ m^−1^·cm ^−1^, low dark toxicity and rapid clearance from the body [16]. Substituted metallophthalocyanines (MPcs) ideally combine all these requirements.

**Figure 1:**
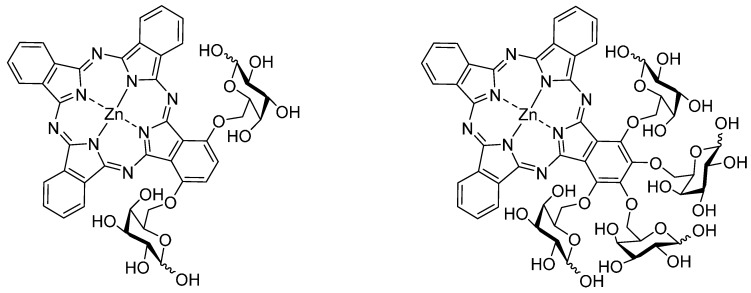
Two examples for AB_3_-type glycoconjugated zinc(II) phthalocyanines [5,23].

One of the most challenging goals when treating cancer is the ability to selectively attack the malignant tumor cells without affecting non-tumorous tissue. For achieving this goal in PDT “second generation PS” have been bound to special carriers [10,17,18] or were modified by polar groups modulating their polarity or simplifying their cellular uptake. Such photosensitizers are named “third generation PS”. For example, Wöhrle showed that the biological activity of Al(III)-phthalocyanines increases with decreasing degree of sulfonation, making the balance between hydrophobic and hydrophilic properties essential for their medical usefulness in PDT [15]. Special carriers for photosensitizers enhancing the specific uptake by tumor cells usually comprise bioactive molecules, such as antibodies, adenoviruses, synthetic peptides or carbohydrates [7,17,18]. Especially glycoconjugated phthalocyanines are promising PS candidates in this respect since the hydroxyl group of the sugar moieties provide hydrophilicity and increase the overall amphiphilicity of the PS [19,20,21]. Due to the increased level of glycolysis and overexpression of sugar transporter proteins in a variety of human carcinomas [22] phthalocyanine sugar conjugates have also been shown to increase their PDT efficacy [5]. Thus, glycosylated phthalocyanines portend being ideal PS for PDT. Figure 1 shows two examples of recently synthesized glycoconjugated phthalocyanines [5,23].

## 2. Results and Discussion

In continuation of our efforts to establish effective third generation PS for PDT [8,23,24,25,26,27,28,29,30,31], we synthesized and fully characterized two new water-soluble, β-d-glucose bearing, AB_3_-type zinc(II) phthalocyanines here. These new conjugates are constructed out of the phthalocyanine core decorated with 1,2,3-triazole-linked methyl glycosides. In 2012, Soares and co-worker showed that such non-symmetrical AB_3_-type ZnPcs exhibit higher cellular uptake rates than the corresponding symmetrical tetra- and octasubstituted counterparts [2,6]. The triazole linker between the phthalocyanine core and the sugar moieties is chosen here for it provides as much to the photosensitizer’s high stability and biocompatibility as minimizing its dark toxicity [32]. Methyl β-d-glucopyranside is used as sugar part in order to prevent the formation of α,β-mixtures upon deprotection of the glycoconjugated phthalocyanines. The 2-methoxyethoxymethyl protecting group (MEM-) is used for the protection of the hydroxyl groups of the carbohydrate moieties for it is as stable under to the harsh basic conditions during the formation of the zinc(II)phthalocyanines and it is gently detached under mild acidic conditions afterwards.

**Scheme 1:**
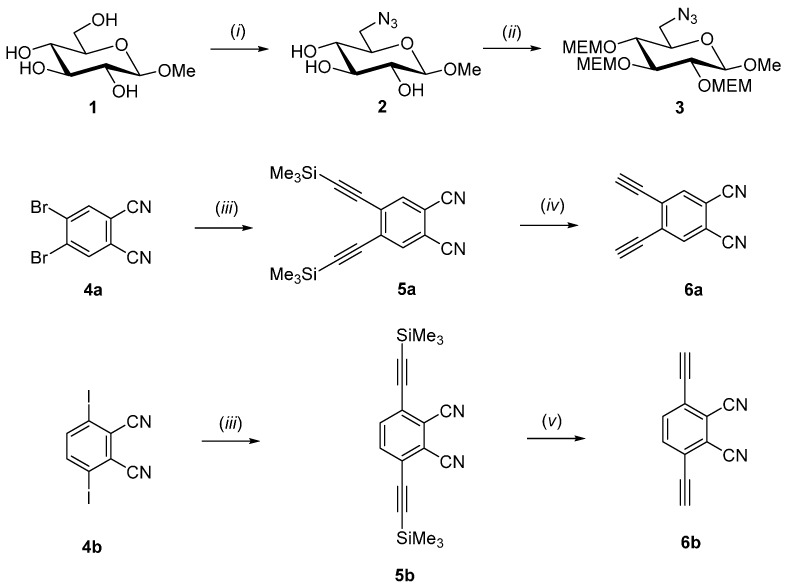
Synthesis of compounds **3** and **6**. Reagents and conditions: (*i*) ref. [33,34]. (*ii*) 2-methoxyethoxymethyl chloride (MEM-Cl), *N*,*N*-diisopropylethylamine (DIPEA), dry DCM, 40 °C, 14 h, 79% **3**; (*iii*) trimethylsilylacetylene, CuI, Pd(PPh_3_)_4_, Et_3_N, 75% **5a**, 72% **5b**; (*iv*) KF in ethanol, 30 min., room temperature, 92% **6a**; (*v*) HF in pyridine, THF, 12 h room temperature, 93% **6b**.

**Scheme 2:**
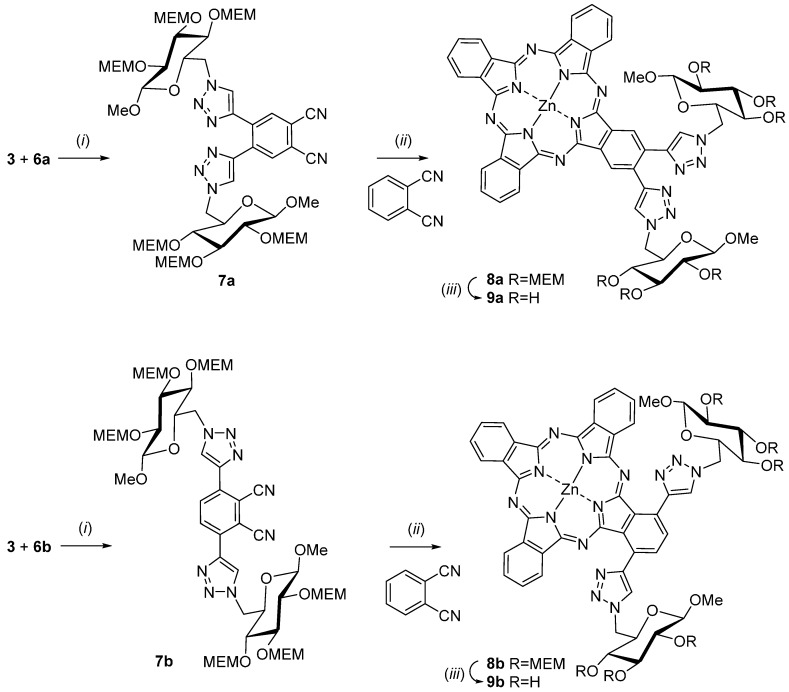
Synthesis of Zn(II)Pc **9a** and **9b**. Reagents and conditions: (*i*) CuI, *N*,*N*,*N*′,*N*′,*N*′′-pentamethyldiethylenetriamine, THF, 14 h, room temperature, 86% **7a**, 84% **7b**; (*ii*) ZnCl_2_, *n*-pentanol, DBU, 24 h, 90–140 °C, 28% **8a**, 47% **8b**; (*iii*) acetyl chloride, methanol, 40 h, room temperature, 98% **9a**, 96% **9b**.

Scheme 1 and Scheme 2 summarize the synthesis of the two novel AB_3_-type zinc(II)-phthalocyanines. The synthesis started with the preparation of MEM-protected methyl 6-azido-6-deoxy-glucoside **3** which was needed for the subsequent 1,3-dipolar cycloaddition reaction with appropriate phthalonitriles **6a** and **6b** (see Scheme 2). Treatment of commercially available methyl β-d-glucopyranoside **1** with *p*-toluenesulfonylchloride in dry pyridine followed by nucleophilic substitution of the tosylate group with sodium azide gave known 6-azido-glucopyranoside **2** [33,34]. The attachment of the glucose moiety to the phthalocyanine core via a 1,2,3-triazole linker at its position 6 was chosen because we had previously shown that glyconjugated phthalocyanines which are connected over position 6 of the sugar have a smaller tendency to form intermolecular aggregates via π-stacking in solution. Such π-stacking aggregates of phthalocyanines are highly detrimental in PDT since they lead to impaired light absorption properties [35]. Therefore, π-stacking aggregation in solution as low as possible is an inevitable prerequisite to use phthalocyanines as photosensitizer in PDT. Aggregated phthalocyanines significantly decrease their photosensitizing ability by self-quenching instead generating high quantum yields of ^1^O_2_.

The choice of the right hydroxyl protecting group on the sugar moieties constitutes a delicate venture in the synthesis of glycosylated phthalocyanines because the harsh basic conditions necessary for the synthesis of the phthalocyanine ring from suitable phthalonitriles and the ability to remove the protecting group under mild conditions not affecting the phthalocyanine or sugar moieties, limit the number of feasible candidates. We chose the 2-methoxyethoxymethyl protecting group (MEM) for it provided the distinct properties needed for our synthesis. Therefore, we prepared the MEM protected sugar **3** by treatment of **2** with 2-methoxyethoxymethyl chloride in dry dichlormethane to afford compound **3** in 79% yield (Scheme 1).

The synthesis of the two bisalkynyl precursors **6** essentially followed the route previously published by Jurícek [36]. First, 4,5-dibromo- **4a** [37,38] and 3,6-diiodophthalonitrile **4b** [37,38] were condensed with trimethylsilylacetylene in a palladium catalyzed Sonogashira coupling [39]. Next, the intermediates **5a** and **5b** were protodesilylated with KF for **5a** and with HF in pyridine for **5b**, respectively, to afford 4,5-bis(ethynyl)phthalonitril **6a** in 92% yield and 3,6-bis(ethynyl)phthalonitril **6b** in 93% yield (Scheme 1).

Next, the syntheses of the glycoconjugated phthalonitriles **7** were achieved by copper(I)-catalyzed cycloaddition (Click reaction) [40] of alkynes **6** and the azide **3**. In detail, the Click reaction was carried out in dry THF with copper(I)iodide as catalyst and *N*,*N*,*N*′,*N*′,*N*′′-pentamethyldiethylenetriamine as the base. Despite the fact that the steric strain during the formation of the triazol ring in phthalonitril **7a** appears to be much higher compared to compound **7b** both Click reactions proceeded smoothly and gave similar yields of 86% for **7a** and 84% for **7b**, respectively. Instead of first converting phthalonitriles **7** into the corresponding AB3-type phthalocyanines and then coupling **3** to the intermediates we chose the inverse sequence here because it was known that the formation of unwanted phthalocyanine isomers of the A4-, A2B2- and AB3 type is diminished when sterically more demanding phthalonitriles such as **7** are used [41,42]. Thus, final purification of the desired AB_3_ type phthalocyanines is prodigiously facilitated.

The preparations of the two zinc(II) phthalocyanines **8** were accomplished using the general method of Tomoda for preparation of phthalocyanines from phthalonitriles [43]. Thus, a solution of the glycoconjugated phthalonitrile precursors **7a** and **7b**, respectively, zinc(II) chloride and an excess of phthalonitrile (11.0 mol equivalents) was heated in *n*-pentanol to 90 °C. The large excess of phthalonitrile was inevitable for obtaining the desired AB_3_-type phthalocyanines in good yields [41,42]. We found that an eleven-fold excess of phthalonitrile over **7** was suited best in this case. Next, DBU was added and the reaction mixtures were heated to 140 °C for 24 h. Purification of MEM-protected glycoconjugated phthalocyanines **8a** and **8b** could be achieved by simple column chromatography on silica gel due to the fact that the only byproduct in both cases was significantly less polar unsubstituted phthalocyanine. The phthalocyanines **8a** and **8b** were obtained in 28% and 47% yield, respectively.

**Figure 2:**
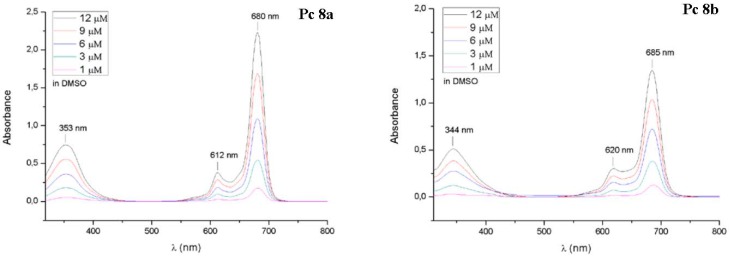
UV-Vis spectra of glycoconjugated phthalocyanine **8a** and **8b** in DMSO at various concentrations.

The UV-Vis spectra of phthalocyanines **8** are shown in Figure 2. The UV-Vis spectra were measured in DMSO at various concentrations (1–12 µM) and display very typical UV-Vis spectra for non-aggregated phthalocyanines with sharp Q-Bands (680–685 nm), vibronic Bands (612–620 nm) and B-Bands (344–353). The NMR spectra of compounds **8a** and **8b** can be found in the supplement. Due to π-stacking aggregation and protonation in CDCl_3_ the NMR spectra of **8a** and **9a** were measured in DMF-*d*_7_ at 100 °C.

**Figure 3:**
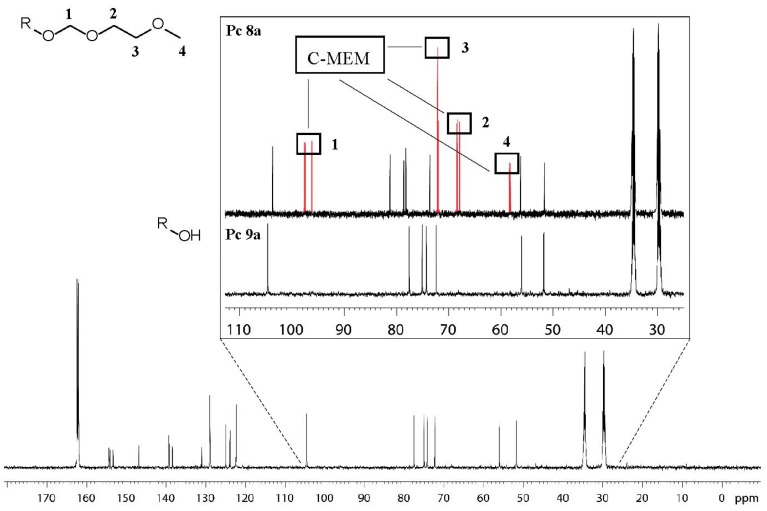
^13^C-NMR spectrum of target compound **9a** measured in DMF-*d*_7_ at 100 °C. The enlarged box shows the disappearance of the ^13^C-Signals after deprotection. Box at the top: MEM protected Pc **8a**. Box at the bottom: deprotected Pc **9a**. R: Glycoconjugated phthalocyanine **9a**.

Finally, the MEM groups in the two phthalocyanines **8** were cleanly cleaved off by simply stirring solutions of the respective phthalocyanines in dry solutions of HCl in methanol according to the procedure previously published by Amano *et al.* [44] (Figure 2). Other deprotecting conditions commonly used for MEM protecting groups such as acetic acid, trifluoroacetic acid, tetrabromomethane, ZnBr_2_ or trimethylsilyltriflate [45,46,47,48] only lead to decomposition or resulted in no reaction at all in our cases. In order to avoid protonated species after the deprotection step, neutralization of the methanolic solution with basic Dowex MWA-1 ion exchange resin was necessary. During neutralization the color of the solution changed from deep green to blue which was indicative for the presence of protonated phthalocyanines in the acid methanolic solution. Similar protonation effects had also been previously observed for other phthalocyanines [49]. Figure 3 shows the ^13^C-NMR spectra of compound **9a**, highlighting the disappearance of the ^13^C signals of the MEM protecting group and thus, showing the clean deprotection of compounds **8** under the chosen conditions. Thus, phthalocyanines **9a** and **9b** were obtained without further purification in virtually quantitative yields.

**Figure 4:**
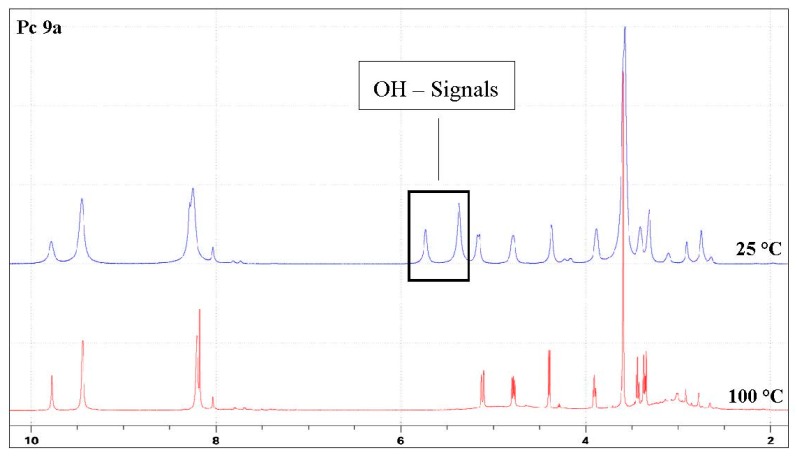
A comparison of the ^1^H-NMR spectra of Pc **9a** measured in DMF-*d*_7_ at 25 °C and 100 °C. The hydroxyl protons of the sugar disappear at 100 °C. Due to the lower aggregation of the phthalocyanines at high temperatures, all non-hydroxyl protons are sharper at 100 °C.

In order to show that both deprotected phthalocyanines **9** are predominantly monomers in solution and indeed do not tend to form π-stacking complexes we measured their ^1^H-NMR spectra in deutero-DMF at room temperature and 100 °C. Figure 4 exemplarily shows these NMR spectra for compound **9a**. The spectra of compound **9b** can be found in the supplement. It was evident from the NMR spectra that both glycoconjugated phthalocyanines **9** are monomeric in solution since no significant changes in the spectra, except for a line sharpening and the disappearance of the signals of the hydroxyl groups due to H/D exchange, could be observed at elevated temperatures. A similar behavior has previously been reported for other phthalocyanines and was attributed to the tendency of phthalocyanines to form complexes to a lesser extent at elevated temperatures or at low concentration [50,51].

**Figure 5:**
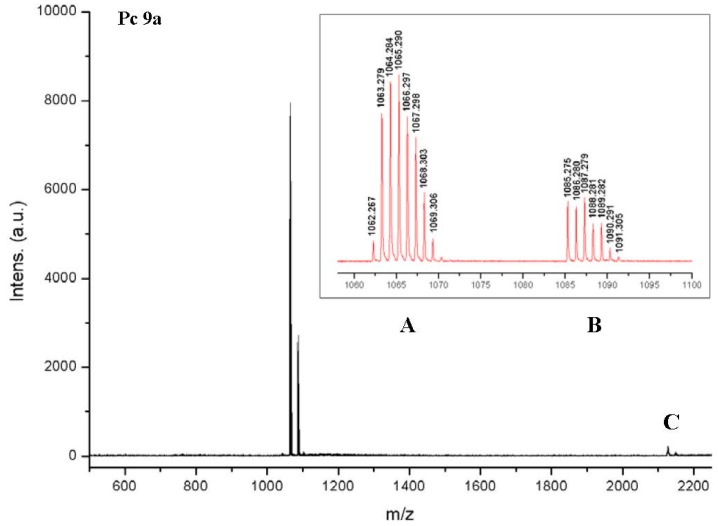
MALDI-TOF spectrum of Pc **9a**. The isotopic pattern shows that the main peak (**A**) is an overlay of [M]^+^ and [M + H]^+^. The smaller peak (**B**) shows the Na adduct of **9a**, revealing the correct isotopic pattern of [M + Na]^+^. C shows the dimer [2M]^+^ and [2M + Na]^+^ of **9a**.

The MALDI-TOF spectrum of **9a** also showed that the deprotection of **8a** was complete (Figure 5). A small amount of the dimer of **9a** could be detected in the mass spectrum though. However, it remained unclear whether such a dimer had formed in solution or during ionization in the MS spectrometer. Similarly, the MALDI-TOF spectrum of compounds **9b** which can be found in the supplement show only the monomer and a small amount of the corresponding dimer.

**Figure 6:**
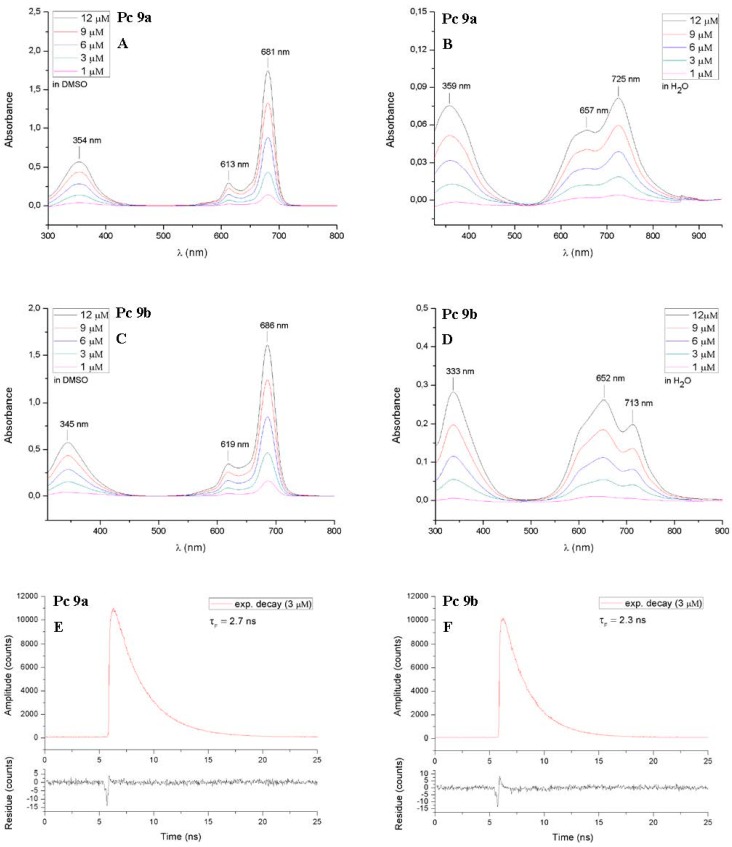
Electronic absorbance spectra of the glycoconjugated phthalocyanines **9a** and **9b**, measured in DMSO (**A**,**B**) and water (**B**,**C**) at various concentrations (1–12 µM). In DMSO, the phthalocyanines show typical non-aggregated UV-Vis spectra with a sharp and intense Q-Band in the red visible light and a Soret band at about 350 nm. In water, both phthalocyanines show aggregation behavior. Their Q-Bands are broadened, much weaker and split into two bands. Furthermore the fluorescence lifetime decay curves of these two phthalocyanines are shown on the bottom of this scheme (**E**,**F**) (solvent: DMSO c = 3 µM).

The UV-Vis spectra of ZnPc **8** and **9** were recorded at increasing concentrations (1, 3, 6, 9 and 12 µM) in DMSO. Compounds **9a** and **9b** were also measured in water (Figure 6). Table 1 summarizes photophysical properties of the glycoconjugated pthalocyanins **8** and **9** which showed the typical electronic absorption spectra for non-aggregated phthalocyanines. Regardless of the concentration, the Q-bands in the red visible light region (680–686 nm), the vibronic bands (612–619 nm) and the B-bands (345–354 nm) were sharp and intense in DMSO. For DMSO being an aprotic, dipolar solvent with the tendency to bind axially to the zinc(II) phthalocyanine aggregation of phthalocyanines is drastically reduced in this solvent [35] The position of the Q-bands depend on the position, number and nature of substituents bound to the phthalocyanine core [52,53] We found the same behavior for the glycoconjugated phthalocyanines described here since the Q-bands of compounds **8b** and **9b** being substituted at the non-peripherally positions (α-position) appear at longer wavelength compared to their peripherally substituted (β-position) constitutional isomers **8a** and **9a**.

**Table 1:** Basic photophysical properties of the phthalocyanines **8a**, **8b** and **9a**, **9b** measured in DMSO.

Compound	λ_max_ (nm (log ε))	λ_em_ (nm) ^a^	τ_F_ (ns) ^b^
**8a**	353 (4.79), 612 (4.50), 680 (5.27)	692	2.6
**8b**	344 (4.63), 620 (4.40), 685 (5.05)	700	2.3
**9a**	354 (4.67), 613 (4.39), 681 (5.16)	693	2.7
**9b**	345 (4.68), 619 (4.46), 686 (5.13)	699	2.3

^a^ Emission spectra: λ_exc_ = 633 nm, ^b^ fluorescence lifetimes.

In general, the absorption properties of a dissolved phthalocyanine are closely mingled with their tendency of forming aggregated species such as dimers. Strong aggregation causes a decrease of the intensity and a broader blue shifted shape of the Q-band. The reason for the hypsochromic shift is the formation of H-type aggregates (hypsochromic aggregates) which are predominately formed in aqueous solutions. Structurally it can be seen as sandwich shaped π-π-stacking [54].

In contrast to the well-defined UV-Vis spectra measured in DMSO the absorption spectra of phthalocyanines **9a** and **9b** in water showed broadened and alleviated Q-bands, due to aggregation. However it must be pointed out that in the case of phthalocyanine **9a** the maximum of the partially divided Q-band is bathochromic shifted to 725 nm instead of the hypsochromic shift usually seen in water. A reason for this may be accounted to the formation of J-type aggregated phthalocyanines which leads to a bathochromic shift of the Q-band in the UV-Vis spectra. Contrary to the sandwich shaped π-π-stacking in H-aggregates, in J-type aggregation the phthalocyanine monomers form slipped cofacial side-by-side aggregates [55]. We suggest that the nitrogen atoms of the triazole moiety in one phthalocyanine molecule coordinates with the zinc(II) metal ion of another phthalocyanine molecule (N → Zn^2+^ bonds). This rather rare behavior leading to J-aggregates has already been described for nitrogen [56,57] and oxygen [54,58,59] substituted phthalocyanines. The finding that the formation of J-aggregates only occurred in the UV-Vis spectra of the peripherally substituted phthalocyanine **9a** can be explained by the better accessibility of the triazole nitrogen atoms due to its greater proximity from the zinc(II) phthalocyanine compared to the closer proximity in **9b**. The fluorescence lifetime decay curves of all the phthalocyanines **8** and **9** are mono-exponential which, in turn leads to the suggestion that no aggregates are present in solution [17]. The fluorescence lifetimes τ_F_ of compounds **8** and **9** (Table 1) are well within the range of most ZnPcs and the τ_F_ values of the two peripherally substituted phthalocyanines **8a** and **9a** are slightly higher than their structural isomers **8b** and **9b**. Finally, it has to be pointed out that the deprotection of the MEM groups, does not influence the photophysical properties in DMSO at all. Neither the absorption maxima λ_max_ nor the fluorescence lifetimes τ_F_ differ between phthalocyanines **8** and **9**.

## 3. Experimental Section

### 3.1. General Information

All starting materials and reagents were purchased from Sigma-Aldrich (Taufkirchen, Germany), ABCR GmbH (Karlsruhe, Germany) and GLYCON Biochemicals GmbH (Luckenwalde, Germany) and were of the highest purity available. All reactions were carried out under anhydrous conditions using flame dried glassware in anhydrous, freshly distilled solvents, unless otherwise noted. TLC was performed on Polygram SIL G/UV_254_ plastic plates (Macherey-Nagel, Düren, Germany), precoated with 0.2 mm thickness of silica gel containing fluorescent indicator. Silica gel 60 (particle size 0.04–0.063 mm) was used for column chromatography. Melting points were determined with a Büchi Melting Point M-560 apparatus (Büchi, Essen, Germany). NMR spectra were measured on an Avance 400 or on a AMX 600 spectrometer (Bruker, Karlsruhe, Germany). The spectra in DMF-*d*_7_ were measured at 25 °C and 100 °C. UV-Vis spectra were recorded on a Lambda 35 instument (Perkin Elmer, Waltham, MA, USA) using a 1 cm quartz cuvette. Elemental analyses were performed on a Euro EA Analyzer (HEKAtech, Wegberg, Germany) using sulfanylamide as standard. Optical rotation measurements were obtained with a Perkin Elmer Model 341 polarimeter. High resolution ESI-TOF mass spectra were measured on a Bruker Daltonics Maxis G4. MALDI-TOF spectra were measured on a Bruker Daltonics Apex 2 using 2,5-dihydroxybenzoic acid (DHB) or *trans*-2-[3-(4-*tert*-butylphenyl)-2-methyl-2- propenylidene]malononitrile (DCTB) as matrix.

### 3.2. Syntheses

*Methyl-6-azido-6-deoxy-2,3,4-tri-O-methoxyethoxymethyl-β-d-glucopyranoside* (**3**): Under an atmosphere of nitrogen, a solution of methyl 6-azido-6-deoxy-*β*-d-glucopyranoside (**2**, 1.5 g, 6.8 mmol, [35]) and *N*,*N*-diisopropylethylamine (DIPEA, 10.7 mL, 61.2 mmol) in dry DCM (80 mL) was cooled to −5 °C. 2-Methoxyethoxymethyl chloride (7 mL, 61.2 mmol) was slowly added over 30 min with stirring at −5 °C. The resulting yellow solution was heated to 40 °C and stirred for 16 h until TLC (petroleum ether/ethyl acetate 1:1) indicated the complete consumption of the starting materials. The mixture was diluted with stirring with DCM (50 mL) and water (100 mL). The organic phase was separated and the aqueous phase extracted three times with DCM. The combined organic phases were washed with 100 mL of saturated aqueous sodium bicarbonate and water, dried over sodium sulfate, filtered and concentrated under reduced pressure. Chromatography (petroleum ether/ethyl acetate 2:3) of the residue gave **3** (2.60 g, 79%) as a colorless oil. ${\left[\text{α}\right]}_{D}^{25}$ +4.7 (*c* 1.0 CHCl_3_); IR (ATR) ν_max_ 2882, 2096 cm^−1^; ^1^H-NMR (CDCl_3_, 400 MHz) δ 4.93–4.76 (6H, m, OC*H*_2_O-MEM), 4.21 (1H, d, *J*_1,2_ = 7.7 Hz, H-1), 3.78–3.69 (5H, m, CH_2_OC*H*_2_C*H*_2_-MEM), 3.61–3.35 (25H, m, H-2, H-3, H-4, H-5, H-6a, H-6b, OC*H*_3_, CH_2_OC*H*_2_C*H*_2_-MEM, CH_2_OC*H*_3_-MEM); ^13^C-NMR (CDCl_3_, 100 MHz) δ 103.8 (CH, C-1), 97.8 (CH_2_, O*C*H_2_O-MEM), 97.5 (CH_2_, O*C*H_2_O-MEM), 97.4 (CH_2_, O*C*H_2_O-MEM), 81.0 (CH, C-3), 78.0 (CH, C-2)*, 77.9 (CH, C-4)*, 74.9 (CH, C-5)*, 72.0 (CH_2_, *C*H_2_OCH_3_-MEM), 71.9 (CH_2_, *C*H_2_OCH_3_-MEM), 71.9 (CH_2_, *C*H_2_OCH_3_-MEM), 68.4 (CH_2_, O*C*H_2_(CH_2_)_3_CH_3_), 68.2 (CH_2_, CH_2_OCH_2_*C*H_2_-MEM), 67.9 (CH_2_, CH_2_OCH_2_*C*H_2_-MEM), 59.2 (CH_3_, CH_2_O*C*H_3_-MEM), 59.2 (CH_3_, CH_2_O*C*H_3_-MEM), 59.2 (CH_3_, CH_2_O*C*H_3_-MEM), 57.0 (CH_3_, O*C*H_3_), 51.8 (CH_2_, C-6); FABMS *m/z* 506 [M+Na]^+^ ; HRESIMS *m/z* 506.23236 (calcd for C_19_H_37_N_3_O_11_Na, 506.23203); anal. C 51.83, H 8.25, N 7.55, calcd for C_24_H_45_N_3_O_11_, C 52.26, H 8.22, N 7.62. * Signals can be interchanged.

*4,5-Bis(trimethylsilylethynyl)phthalonitrile* (**5a**): Under an atmosphere of nitrogen, **4a** [36] (1.45 g, 5.1 mmol), CuI (387 mg, 2.0 mmol) and Pd(Ph_3_)_4_ (360 mg, 0.6 mmol) were suspended in dry trimethylamine (100 mL). The mixture was cooled to 0 °C and trimethylsilylacetylene (1.9 mL, 13.5 mmol) was slowly added. The reaction mixture was heated to 60 °C for 72 h until TLC (dichlormethane/petroleum ether 2:3) showed complete consumption of the starting materials. The reaction mixture was cooled to room temperature, filtered and the filtrate was concentrated under reduced pressure. Chromatography (dichloromethane/petroleum ether 2:3) of the residue and crystallization from ethanol gave compound **5a** (1.22 g, 75%) as colorless needles. mp 150–151 °C (ethanol); IR (KBr) ν_max_ 2964, 2237, 1808 cm^−1^; ^1^H-NMR (CDCl_3_, 400 MHz) δ 7.83 (2H, s, *H*-C), 0.29 (18H, s, C*H*_3_); ^13^C-NMR (CDCl_3_, 100 MHz) δ 136.9 (CH, C-Aryl), 130.9 (C, C-Aryl), 114.6 (C, *C*N)*, 114.4 (C, Aryl-*C*-CN)*, 107.4 (C, C-Alkyne), 99.7 (C, *C*Si), -0.3 (CH_3_, CH_3_); EIMS *m/z* 320 [M]^+^; anal. C 67.31, H 6.27, N 8.77, calcd for C_18_H_20_N_2_Si_2_, C 67.45, H 6.29, N 8.74. * Signals can be interchanged.

*3,6-Bis(trimethylsilylethynyl)phthalonitrile* (**5b**): Under an atmosphere of nitrogen, **4b** [38] (4.4 g, 11.5 mmol), CuI (3.5 g, 18.5 mmol) and Pd(Ph_3_)_2_Cl_2_ (350 mg, 0.5 mmol) were suspended in dry trimethylamine (200 mL). The reaction mixture was cooled to 0 °C and trimethylsilylacetylene (1.9 mL, 13.5 mmol) was slowly added. The reaction mixture was stirred at room temperature for 16 h until TLC (dichlormethane/petroleum ether 2:3) indicated the complete consumption of the starting materials. The mixture was filtrated and the filtrate was concentrated under reduced pressure. Chromatography (dichloromethane/petroleum ether 2:3) of the residue and crystallization from ethanol gave **5b** (2.7 g, 72%) as colorless needles. mp 138–139 °C (ethanol); IR (KBr) ν_max_ 3080, 2959, 2901, 2360, 2234, 2156, 1972 cm^−1^; ^1^H-NMR (CDCl_3_, 400 MHz) δ 7.66 (2H, s, *H*-C), 0.29 (18H, s, C*H*_3_); ^13^C-NMR (CDCl_3_, 100 MHz) δ 135.9 (CH, C-Aryl), 128.2 (C, C-Aryl), 119.9 (C, Aryl-*C*-CN), 114.4 (C, *C*N), 107.8 (C, C-Alkyne), 99.2 (C, *C*Si), -0.3 (CH_3_, CH_3_); EIMS *m/z* 320 [M]^+^; HRESIMS *m/z* 343.10550 (calcd for C_18_H_20_N_2_Si_2_Na, 343.10572); anal. C 67.18, H 6.26, N 8.76, calcd for C_18_H_20_N_2_Si_2_, C 67.45, H 6.29, N 8.74. * Signals can be interchanged.

*4,5-Bis(ethynyl)phthalonitril* (**6a**): A solution of **5a** (2.0 g, 6.2 mmol) and potassium fluoride (1.45 g, 25 mmol) in ethanol (150 mL) was stirred at room temperature for 30 min until TLC (ethyl acetate/petroleum ether 1:4) showed the complete consumption of the starting material. Concentration of the mixture under reduced pressure and chromatography (ethyl acetate/petroleum ether 1:4) of the residue gave **6a** (1.01 g, 92%) as colorless amorphous solid. ^1^H-NMR (CDCl_3_, 400 MHz) δ 7.90 (2H, s, *H*-C), 3.69 (2H, s, *H*-alkyne); ^13^C-NMR (CDCl_3_, 100 MHz) δ 137.2 (CH, C-Aryl), 130.5 (C, C-Aryl), 115.4 (C, *C*N)*, 114.3 (C, Aryl-*C*-CN)*, 88.3 (C, C-Alkyne), 78.8 (CH, C-Alkyne); * Signals can be interchanged. The characterization is in agreement with the literature [60].

*3,6-Bis(ethynyl)phthalonitril* (**6b**): Under an atmosphere of nitrogen, **5b** (100 mg, 0.31 mmol) was dissolved in dry THF (30 mL) and cooled to −10 °C. HF in pyridine (0.4 mL, 15.6 mmol) was slowly added to the stirred mixture and stirring was continued for 1 h at −10 °C and for 12 h at room temperature until TLC (ethyl acetate/petroleum ether 1:4) showed the complete consumption of the starting material. The reaction mixture was diluted with ethyl acetate (50 mL), washed with saturated aqueous sodium bicarbonate (60 mL) and water (60 mL), dried over sodium sulfate and filtered. Concentration of the filtrate under reduced pressure and chromatography (ethyl acetate/petroleum ether 1:4) of the residue gave **6a** (50 mg, 93%) as colorless amorphous solid. ^1^H-NMR (CDCl_3_, 400 MHz) δ 7.77 (2H, s, *H*-C), 3.70 (2H, s, *H*-alkyne); ^13^C-NMR (CDCl_3_, 100 MHz) δ 135.2 (CH, C-Aryl), 126.5 (C, C-Aryl), 118.9 (C, Aryl-*C*-CN)*, 112.7 (C, *C*N)*, 87.1 (C, C-Alkyne), 77.0 (CH, C-Alkyne); EIMS *m/z* 176 [M]^+^ ; HRESIMS *m/z* 343.10550 (calcd for C_18_H_20_N_2_Si_2_Na, 343.10572); anal. C 67.18, H 6.26, N 8.76, calcd for C_18_H_20_N_2_Si_2_, C 67.45, H 6.29, N 8.74. * Signals can be interchanged.

*4,5-Bis[1-(6-deoxy-2,3,4-tri-O-methoxyethoxymethyl-1-O-methyl-β-d-glucopyranose-6-yl)1H-1,2,3-triazole-4-yl]phthalonitrile* (**7a**): Under an atmosphere of nitrogen, **3** (970 mg, 2.0 mmol), **6a** (161 mg, 0.92 mmol), CuI (191 mg, 1.0 mmol) and *N*,*N*,*N*′,*N*′,*N*′′-pentamethyldiethylenetriamine (269 µL, 1.3 mmol) were dissolved in dry THF (20 mL) and the solution was stirred at room temperature for 14 h until TLC (ethyl acetate/methanol 19:1) indicated complete consumption of the starting materials. The green reaction mixture was diluted with 80 mL DCM, washed with 1M aqueous NH_4_Cl solution, dried over sodium sulfate, filtered and concentrated under reduced pressure. Chromatography (ethyl acetate/methanol 19:1) gave **7a** (902 mg, 86%) as a yellow viscous oil. ${\left[\text{α}\right]}_{D}^{25}$ −19 (*c* 1.0 CHCl_3_); IR (ATR) ν_max_ 2889, 2360 cm^−1^; ^1^H-NMR (CDCl_3_, 400 MHz) δ 8.16 (2H, s, *H*-Phenyl), 7.75 (2H, s, *H*-Triazole), 4.98 (2H, dd, *J*_6a,6b_ = 14.4 Hz, *J*_6a,5_ = 2.2 Hz, H-6a), 4.97–4.69 (12H, m, OC*H*_2_O-MEM), 4.35 (2H, dd, *J*_6b,6a_ = 14.5 Hz, *J*_6b,5_ = 8.3 Hz, H-6b), 4.06 (2H, d, *J*_1,2_ = 7.7 Hz, H-1), 3.84 (2H, m, CH_2_OC*H*_2_CH_2_-MEM), 3.70–3.42 (26H, m, H-3, H-5, CH_2_OC*H*_2_C*H*_2_-MEM), 3.35–3.24 (28H, m, H-2, H-4, OC*H*_3_, CH_2_OC*H*_3_-MEM); ^13^C-NMR (CDCl_3_, 100 MHz) δ 142.8 (C, C-Triazole), 135.1 (CH, C-Aryl), 134.3 (C, C-Aryl)*, 125.3 (CH, C-Triazole), 115.0 (C, C-Aryl)*, 114.9 (C, *C*N)*, 103.5 (CH, C-1), 97.7 (CH_2_, O*C*H_2_O-MEM), 96.9 (CH_2_, O*C*H_2_O-MEM), 96.4 (CH_2_, O*C*H_2_O-MEM), 80.5 (CH, C-3)**, 78.1 (CH, C-4)***, 77.4 (CH, C-2)***, 73.1 (CH, C-5)**, 71.7 (CH_2_, *C*H_2_OCH_3_-MEM), 71.6 (CH_2_, *C*H_2_OCH_3_-MEM), 71.6 (CH_2_, *C*H_2_OCH_3_-MEM), 68.2 (CH_2_, O*C*H_2_(CH_2_)_3_CH_3_), 67.9 (CH_2_, O*C*H_2_(CH_2_)_3_CH_3_), 67.5 (CH_2_, O*C*H_2_(CH_2_)_3_CH_3_), 58.9 (CH_3_, CH_2_O*C*H_3_-MEM), 58.8 (CH_3_, CH_2_O*C*H_3_-MEM), 58.8 (CH_3_, CH_2_O*C*H_3_-MEM), 56.9 (CH_3_, O*C*H_3_), 51.6 (CH_2_, C-6); FABMS *m/z* 1166 [M + Na]^+^ ; HRESIMS *m/z* 1165.51355 (calcd for C_50_H_78_N_8_O_22_Na, 1165.51229); anal. C 52.62, H 6.93, N 9.82, calcd for C_50_H_78_N_8_O_22_, C 52.53, H 6.88, N 9.80. *, **, *** Signals can be interchanged.

*3,6-Bis[1-(6-deoxy-2,3,4-tri-O-methoxyethoxymethyl-1-O-methyl-β-d-glucopyranose-6-yl)1H-1,2,3-triazole-4-yl]phthalonitrile* (**7b**): Treatment of **3** (1.5 g, 3.1 mmol), **6b** (248 mg, 1.4 mmol), CuI (295 mg, 1.6 mmol) and *N*,*N*,*N*′,*N*′,*N*′′-pentamethyldiethylenetriamine (323 µL, 1.6 mmol) as described for the preparation of compound **7a** followed by chromatography (ethyl acetate/methanol 30:1) gave **7b** (1.35 g, 84%) as a yellow amorphous solid. [α]^25^_D_ −42 (*c* 1.0 CHCl_3_); IR (ATR) ν_max_ 2885, 2360 cm^−1^; ^1^H-NMR (CDCl_3_, 400 MHz) δ 8.72 (2H, s, *H*-Phenyl), 8.63 (2H, s, *H*-Triazole), 5.17 (2H, dd, *J*_6a,6b_ = 14.5 Hz, *J*_6a,5_ = 2.1 Hz, H-6a), 5.02–4.79 (12H, m, OC*H*_2_O-MEM), 4.49 (2H, dd, *J*_6b,6a_ = 14.5 Hz, *J*_6b,5_ = 8.5 Hz, H-6b), 4.16 (2H, d, *J*_1,2_ = 7.7 Hz, H-1), 3.96 (2H, ddd, CH_2_OC*H*_2_CH_2_-MEM), 3.77–3.50 (26H, m, H-3, H-5, CH_2_OC*H*_2_C*H*_2_-MEM), 3.46–3.35 (28H, m, H-2, H-4, OC*H*_3_, CH_2_OC*H*_3_-MEM); ^13^C-NMR (CDCl_3_, 100 MHz) δ 141.9 (C, C-Triazole), 134.8 (C, C-Aryl), 132.4 (CH, C-Aryl)*, 124.7 (CH, C-Triazole), 116.0 (C, C-Aryl)*, 112.0 (C, *C*N)*, 103.7 (CH, C-1), 98.0 (CH_2_, O*C*H_2_O-MEM), 97.5 (CH_2_, O*C*H_2_O-MEM), 96.3 (CH_2_, O*C*H_2_O-MEM), 80.7 (CH, C-3), 78.4 (CH, C-4)**, 77.7 (CH, C-2)**, 73.5 (CH, C-5), 71.9 (CH_2_, *C*H_2_OCH_3_-MEM), 71.8 (CH_2_, *C*H_2_OCH_3_-MEM), 71.8 (CH_2_, *C*H_2_OCH_3_-MEM), 68.6 (CH_2_, O*C*H_2_(CH_2_)_3_CH_3_), 68.2 (CH_2_, O*C*H_2_(CH_2_)_3_CH_3_), 67.8 (CH_2_, O*C*H_2_(CH_2_)_3_CH_3_), 59.2 (CH_3_, CH_2_O*C*H_3_-MEM), 59.1 (CH_3_, CH_2_O*C*H_3_-MEM), 57.3 (CH_3_, CH_2_O*C*H_3_-MEM), 57.3 (CH_3_, O*C*H_3_), 52.1 (CH_2_, C-6); FABMS *m/z* 1166 [M + Na]^+^; HRESIMS *m/z* 1165.51229 (calcd for C_50_H_78_N_8_O_22_Na, 1165.51229); anal. C 52.39, H 6.92, N 9.78, calcd for C_50_H_78_N_8_O_22_, C 52.53, H 6.88, N 9.80. *,** Signals can be interchanged.

*[2,3-Bis(1-(6-deoxy-2,3,4-tri-O-methoxyethoxymethyl-1-O-methyl-β-d-glucopyranose-6-yl)1H-1,2,3-triazole-4-yl)phthalocyaninato]zinc(II)* (**8a**): under an atmosphere of nitrogen, **7a** (500 mg 0.44 mmol), phthalonitrile (616 mg 4.8 mmol) and anhydrous ZnCl_2_ (656 mg 4.8 mmol) were suspended in freshly distilled *n*-pentanol (10 mL). The reaction mixture was heated to 90 °C and stirred for 30 min. Next DBU (724 µL 4.8 mmol) was added and the solution was stirred at 140 °C for 24 h. The mixture was cooled to room temperature concentrated under reduced pressure. Chromatography (chloroform containg 1.5% methanol and 0.1% triethylamine) followed by a second chromatography (toluene/acetone gradient 2:1 to 1:1 containing 1% triethylamine) gave **8a** (196 mg, 28%) as a blue amorphous solid. IR (KBr) ν_max_ 2885, 2835 cm^−1^; UV (DMSO) λ max (log ε) 353 (4.79), 612 (4.50), 680 (5.27) nm; ^1^H-NMR (DMF-*d*_7_, 400 MHz) δ 9.28–9.26 (6H, m, *H*-PC), 9.12 (2H, s, *H*-Phenyl), 8.20–8.15 (6H, m, *H*-PC), 7.65 (2H, s, *H*-Triazole), 5.07–5.01 (8H, m, OC*H*_2_O-MEM), 4.97 (2H, d, OC*H*_2_O-MEM), 4.90–4.86 (4H, m, H-6a, OC*H*_2_O-MEM), 4.48 (2H, dd, *J*_6b,6a_ = 14.0 Hz, *J*_6b,5_ = 8.4 Hz, H-6b), 4.31 (2H, d, *J*_1,2_ = 7.5 Hz, H-1), 3.96–3.92 (2H, m, CH_2_OC*H*_2_C*H*_2_-MEM), 3.88–3.84 (6H, m, CH_2_OC*H*_2_C*H*_2_-MEM), 3.81–3.79 (4H, m, CH_2_OC*H*_2_C*H*_2_-MEM), 3.78–3.72 (4H, m, H-3, H-5), 3.69 (4H, dd, CH_2_OC*H*_2_C*H*_2_-MEM), 3.63 (4H, dd, CH_2_OC*H*_2_C*H*_2_-MEM), 3.59 (4H, dd, CH_2_OC*H*_2_C*H*_2_-MEM), 3.51 (2H, dd, *J*_4,5_ = 9.1 Hz, *J*_4,3_ = 9.1 Hz, H-4), 3.46–3.43 (14H, m, H-2, OC*H*_3_, CH_2_OC*H*_3_-MEM), 3.41 (6H, s, CH_2_OC*H*_3_-MEM), 3.37 (6H, s, CH_2_OC*H*_3_-MEM); ^13^C-NMR (DMF-*d*_7_, 100 MHz) δ 154.1 (C, C-Aryl), 153.9 (C, C-Aryl), 153.5 (C, C-Aryl), 152.7 (C, C-Aryl), 146.0 (C, C-Triazole), 138.9 (C, C-Aryl), 138.8 (C, C-Aryl), 138.7 (C, C-Aryl), 137.8 (C, C-Aryl), 129.9 (C, C-Aryl), 129.3 (CH, *C*H-PC), 129.3 (CH, *C*H-PC), 129.3 (CH, *C*H-PC), 124.7 (CH, *C*H-Triazole), 123.8 (CH, *C*H-Aryl), 122.6 (CH, C-Aryl), 122.6 (C, C-Aryl), 122.5 (CH, *C*H-PC), 103.7 (CH, C-1), 97.6 (CH_2_, O*C*H_2_O-MEM), 97.5 (CH_2_, O*C*H_2_O-MEM), 96.2 (CH_2_, O*C*H_2_O-MEM), 81.2 (CH, C-3), 78.6 (CH, C-2), 78.2 (CH, C-4), 73.6 (CH, C-5), 72.1 (CH_2_, *C*H_2_OCH_3_-MEM), 72.1 (CH_2_, *C*H_2_OCH_3_-MEM), 72.0 (CH_2_, *C*H_2_OCH_3_-MEM), 68.5 (CH_2_, O*C*H_2_(CH_2_)_3_CH_3_), 68.2 (CH_2_, O*C*H_2_(CH_2_)_3_CH_3_), 67.9 (CH_2_, O*C*H_2_(CH_2_)_3_CH_3_), 58.3 (CH_3_, CH_2_O*C*H_3_-MEM), 58.2 (CH_3_, CH_2_O*C*H_3_-MEM), 58.2 (CH_3_, CH_2_O*C*H_3_-MEM), 56.2 (CH_3_, OCH_3_), 51.6 (CH_2_, C-6); MALDI-MS *m/z* 1614 [M + Na]^+^; HRESIMS *m/z* 818.27218 (calcd for [C_74_H_90_N_14_O_22_ZnNa_2_]^2+^, 818.27150); anal. C 56.28, H 5.73, N 12.31, calcd for C_74_H_90_N_14_O_22_Zn, C 55.80, H 5.69, N 12.31.

*[1,4-Bis(1-(6-deoxy-2,3,4-tri-O-methoxyethoxymethyl-1-O-methyl-β-d-glucopyranose-6-yl)1H-1,2,3-triazole-4-yl)phthalocyaninato]zinc(II)* (**8b**): Treatment of **7b** (500 mg 0.44 mmol), phthalonitrile (616 mg 4.8 mmol), anhydrous ZnCl_2_ (656 mg 4.8 mmol) and DBU (724 µL 4.8 mmol) in freshly distilled *n*-pentanol (10 mL) as described for the preparation of **8a** gave **8b** (329 mg, 47%) as a blue amorphous solid. IR (KBr) ν_max_ 2886, 2830 cm^−1^; UV (DMSO) λ max (log ε) 344 (4.63), 620 (4.40), 685 (5.05) nm; ^1^H-NMR (DMF-*d*_7_, 400 MHz) δ 9.45 (2H, s, *H*-Phenyl), 9.26–9.22 (4H, m, *H*-PC), 8.13–8.12 (2H, m, *H*-PC), 8.04–7.91 (4H, m, *H*-PC), 7.57 (2H, s, *H*-Triazole), 5.17–5.07 (10H, m, H-6a, OC*H*_2_O-MEM), 4.91 (2H, d, OC*H*_2_O-MEM), 4.84 (2H, d, OCH_2_O-MEM), 4.77–4.73 (2H, m, H-6b), 4.27 (2H, d, *J*_1,2_ = 6.9 Hz, H-1), 4.06–4.04 (2H, m, CH_2_OC*H*_2_C*H*_2_-MEM), 3.97–3.91 (8H, m, H-5, CH_2_OC*H*_2_C*H*_2_-MEM), 3.82–3.69 (17H, m, H-3, H-4, CH_2_OC*H*_2_C*H*_2_-MEM), 3.54–3.43 (17H, m, H-2, CH_2_OC*H*_2_C*H*_2_-MEM , CH_2_OC*H*_3_-MEM), 3.32 (6H, s, CH_2_OC*H*_3_-MEM), 3.04 (6H, s, OC*H*_3_); ^13^C-NMR (DMF-*d*_7_, 100 MHz) δ 155.5 (C, C-Aryl)*, 155.2 (C, C-Aryl)*, 153.2 (C, C-Aryl)*, 151.9 (C, C-Aryl)*, 145.1 (C, C-Aryl)*, 140.1 (C, C-Aryl)*, 139.5 (C, C-Aryl)*, 139.4 (C, C-Triazole)*, 134.7 (C, C-Aryl)*, 130.4 (CH, C-Triazole)**, 130.2 (CH, *C*H-PC)**, 130.0 (CH, *C*H-PC)**, 129.8 (CH, *C*H-PC)**, 128.9 (CH, C-Aryl), 126.7 (C, C-Aryl), 123.6 (CH, *C*H-PC), 123.4 (CH, *C*H-PC) 123.1 (CH, *C*H-PC), 104.7 (CH, C-1), 98.8 (CH_2_, O*C*H_2_O-MEM), 98.6 (CH_2_, O*C*H_2_O-MEM), 97.2 (CH_2_, O*C*H_2_O-MEM), 82.4 (CH, C-3), 79.6 (CH, C-4)**, 79.5 (CH, C-2)**, 74.7 (CH, C-5), 73.2 (CH_2_, *C*H_2_OCH_3_-MEM), 73.2 (CH_2_, *C*H_2_OCH_3_-MEM), 72.9 (CH_2_, *C*H_2_OCH_3_-MEM), 69.6 (CH_2_, O*C*H_2_(CH_2_)_3_CH_3_), 69.3 (CH_2_, O*C*H_2_(CH_2_)_3_CH_3_), 68.8 (CH_2_, O*C*H_2_(CH_2_)_3_CH_3_), 59.4 (CH_3_, CH_2_O*C*H_3_-MEM), 59.3 (CH_3_, CH_2_O*C*H_3_-MEM), 59.1 (CH_3_, CH_2_O*C*H_3_-MEM), 56.9 (CH_3_, OCH_3_), 53.3 (CH_2_, C-6); MALDI-MS *m/z* 1591 [M]^+^; HRESIMS *m/z* 1591.57070 (calcd for [C_148_H_182_N_28_O_44_Zn_2_]^2+^, 1591.57183); anal. C 55.81, H 5.79, N 12.34, calcd for C_74_H_90_N_14_O_22_Zn, C 55.80, H 5.69, N 12.31. *,** Signals can be interchanged.

*[2,3-Bis(1-(6-deoxy-1-O-methyl-β-d-glucopyranose-6-yl)1H-1,2,3-triazole-4-yl)phthalocyaninato]-zinc(II)* (**9a**): Under an atmosphere of nitrogen, a solution of **8a** (97 mg, 0.060 mmol) in methanol (12 mL) was cooled to 0 °C and acetyl chloride (250 µL, 3.5 mmol) was slowly added under stirring with a syringe whereupon the solution changed its color from blue to green. The solution was stirred for 40 h at room temperature until TLC (chloroform/methanol 3:1 containing 1% triethylamine) indicated the complete consumption of the starting material. The solution was diluted with 20 mL methanol, neutralized with Dowex MWA-1 ion-exchange and concentrated under reduced pressure to give **9a** (63 mg, 98%) as blue amorphous solid. IR (KBr) ν_max_ 3419, 2916, 2850 cm^−1^; UV (DMSO) λ max (log ε) 354 (4.67), 613 (4.39), 681 (5.16) nm; ^1^H-NMR (DMF-*d*_7_, 400 MHz) δ 9.77 (2H, s, *H*-Phenyl), 9.44 (6H, m, *H*-PC), 8.21 (6H, m, *H*-PC), 8.17 (2H, s, *H*-Triazole), 5.13–5.10 (2H, m, H-6a), 4.78 (2H, dd, *J*_6b,6a_ = 7.3 Hz, *J*_6b,5_ = 7.3 Hz, H-6b), 4.39 (2H, d, *J*_1,2_ = 7.7 Hz, H-1), 3.92–3.89 (2H, m, H-5), 3.61–3.59 (8H, m, H-3, OC*H*_3_), 3.44 (2H, dd, *J*_4,5_ = 9.2 Hz, *J*_4,3_ = 9.2 Hz, H-4), 3.34 (2H, dd, *J*_2,1_ = 8.3 Hz, *J*_2,3_ = 8.3 Hz, H-2); ^13^C-NMR (DMF-*d*_7_, 100 MHz) δ 154.5 (C, C-Aryl), 154.4 (C, C-Aryl), 154.1 (C, C-Aryl), 153.4 (C, C-Aryl), 146.9 (C, C-Triazole), 139.3 (C, C-Aryl), 139.3 (C, C-Aryl), 139.3 (C, C-Aryl), 138.4 (C, C-Aryl), 131.1 (C, C-Aryl), 129.0 (CH, *C*H-PC), 129.0 (CH, *C*H-PC), 128.9 (CH, *C*H-PC), 125.0 (CH, *C*H-Triazole), 124.0 (CH, *C*H-Aryl), 122.4 (CH, *C*H-PC), 122.4 (CH, *C*H-PC), 122.4 (CH, *C*H-PC), 104.6 (CH, C-1), 77.5 (CH, C-3), 75.1 (CH, C-5), 74.3 (CH, C-2), 72.4 (CH, C-4), 56.1 (CH_3_, OCH_3_), 51.8 (CH_2_, C-6); MALDI-MS *m/z* 1062 [Ma]^+^; HRESIMS *m/z* 1063.25767 (calcd for [C_50_H_43_N_14_O_10_Zn]^+^, 1063.25723).

*[1,4-Bis(1-(6-deoxy-1-O-methyl-β-D-glucopyranose-6-yl)1H-1,2,3-triazole-4-yl)phthalocyaninato]-zinc(II)* (**9b**): Treatment a solution of **8b** (107 mg, 0.067 mmol) in methanol (15 mL) with acetyl chloride (300 µL, 4.2 mmol) and workup as decribed for the preparation of compound **9a** gave **9b** (69 mg, 96%) as a blue amorphous solid. IR (KBr) ν_max_ 3385, 2921 cm^−1^; UV (DMSO) λ max (log ε) 345 (4.68), 619 (4.46), 686 (5.13) nm; ^1^H-NMR (DMF-*d*_7_, 400 MHz) δ 10.04 (2H, s, *H*-Phenyl), 9.17–9.13 (4H, m, *H*-PC), 8.89 (2H, m, *H*-PC)*, 8.62 (2H, s, *H*-Triazole)*, 8.11–8.10 (6H, m, *H*-PC), 5.54–5.52 (2H, m, H-6a), 5.13 (2H, dd, *J*_6b,6a_ = 14.4 Hz, *J*_6b,5_ = 8.5 Hz, H-6b), 4.32 (2H, d, *J*_1,2_ = 7.6 Hz, H-1), 4.22–4.20 (2H, m, H-5), 3.65–3.63 (4H, m, H-3, H-4), 3.37 (2H, m, H-2), 3.21 (6H, s, OC*H*_3_); ^13^C-NMR (DMF-*d*_7_, 100 MHz) δ 155.2 (C, C-Aryl), 154.5 (C, C-Aryl), 145.0 (C, C-Triazole), 139.0 (C, C-Aryl), 138.6 (C, C-Aryl), 129.9 (CH, *C*H-PC), 129.9 (CH, *C*H-PC), 129.6 (CH, *C*H-PC), 129.3 (CH, *C*H-Triazole), 128.3 (C, C-Aryl), 122.6 (CH, *C*H-PC), 122.5 (CH, *C*H-PC), 121.9 (CH, *C*H-PC), 104.7 (CH, C-1), 77.6 (CH, C-3)**, 75.3 (CH, C-2)**, 74.4 (CH, C-4)**, 72.9 (CH, C-5)**, 55.9 (CH_3_, OCH_3_), 52.6 (CH_2_, C-6); MALDI-MS *m/z* 1063 [M + H]^+^; HRESIMS *m/z* 1063.25910 (calcd for [C_50_H_43_N_14_O_10_Zn]^+^, 1063.25725). *,** Signals can be interchanged. ^13^C: Some quartary signals are missing.

## 4. Conclusions

In summary, two novel water-soluble, glycoconjugated phthalocyanines were synthesized and fully characterized. The synthesis was guided by the requirements for simplicity, feasibility of scaleup and the ability to prepare a variety of compounds from the same carbohydrate precursors. The MEM protecting group for blocking all hydroxyls of the sugar moieties, hitherto not yet used for Pc syntheses, was shown to be fully adequate for the preparation of glycoconjugated phthalocyanines. All phthalocyanines described herein were fully characterized by means of MALDI-TOF spectrometry and temperature dependent NMR spectroscopy. The Q-bands of all phthalocyanines are in the red visible light region (>680 nm) and the molar extinction coefficents ε_max_ were > 10^5^ m^−1^ cm ^−1^. The Q-band of the non-peripherally substituted Pc **9a** was found to be red shifted compared to the Q-band of its constitutional isomer **9b**, whereas the fluorescence lifetime τ_F_ of **9b** (2.7 ns) was determined to be higher than the τ_F_ of **9a**. The photophysical results revealed that the water-soluble AB_3_-type Zn(II)Pcs are suitable photosensitizer candidates for medical applications in photodynamic therapy.

## Supplementary Material

The spectroscopic data of all novel compounds presented in this article, are listed in the enclosed supplementary material. The data include ^1^H-NMR- and ^13^C-NMR-, MALDI-TOF, UV-Vis spectra and the decay curves of the fluorescence lifetime measurements can be accessed at: http://www.mdpi.com/1420-3049/20/10/18367/s1.

## References

[1] McKeown N.B. (1998). Phthalocyanine Materials: Synthesis, Structure and Function.

[2] Soares A.R.M., Tomé J.P.C., Neves M.G.P.M.S., Tomé A.C., Cavaleiro J.A.S., Torres T. (2009). Synthesis of water-soluble phthalocyanines bearing four or eight d-galactose units. Carbohydr. Res..

[3] Liu J.-Y., Lo P.-C., Fong W.-P., Ng D.K.P. (2009). Effects of the number and position of the substituents on the *in vitro* photodynamic activities of glucosylated zinc(II) phthalocyanines. Org. Biomol. Chem..

[4] Kimani S.G., Shmigol T.A., Hammond S., Phillips J.B., Bruce J.I., MacRobert A.J., Malakhov M.V., Golding J.P. (2013). Fully Protected Glycosylated Zink (II) Phthalocyanine Shows High Uptake and Photodynamic Cytotoxicity in MCF.7 Cancer Cells. Photochem. Photobiol..

[5] Ribeiro A.O., Tomé J.P.C., Neves M.G.P.M.S., Tomé A.C., Cavaleiro J.A.S., Iamamoto Y., Torres T. (2006). [1,2,3,4-Tetrakis(α/β-d-galactopyranos-6-yl)phthalocyaninato]zinc(II): A water-soluble phthalocyanine. Tetrahedron Lett..

[6] Soares A.R.M., Neves M.G.P.M.S., Tomé A.C., Iglesias-de la Cruz M.C., Zamarrón A., Carrasco E., González S., Cavaleiro J.A.S., Torres T., Guldi D.M. (2012). Glycophthalocyanines as Photosensitizers for Triggering Mitotic Catastrophe and Apoptosis in Cancer Cells. Chem. Res. Toxicol..

[7] Choi C.-F., Huang J.-D., Lo P.-C., Fong W.-P., Ng D.K.P. (2008). Glycosylated zinc(II) phthalocyanines as efficient photosensitisers for photodynamic therapy. Synthesis, photophysical properties and *in vitro* photodynamic activity. Org. Biomol. Chem..

[8] Alvarez-Micó X., Calvete M.J.F., Hanack M., Ziegler T. (2006). The first example of anomeric glycoconjugation to phthalocyanines. Tetrahedron Lett..

[9] Antoni P.M., Naik A., Albert I., Rubbiani R., Gupta S., Ruiz-Sanchez P., Munikorn P., Mateos J.M., Luginbuehl V., Thamyongkit P. (2015). (Metallo)porphyrins as Potent Phototoxic Anti-Cancer Agents after Irradiation with Red Light. Chem. Eur. J..

[10] DeRosa M.C., Crutchley R.J. (2002). Photosensitized singlet oxygen and its applications. Coord. Chem. Rev..

[11] Dąbrowski J.M., Arnaut L.G., Pereira M.M., Urbańska K., Simões S., Stochel G., Cortes L. (2012). Combined effects of singlet oxygen and hydroxyl radical in photodynamic therapy with photostable bacteriochlorins: Evidence from intracellular fluorescence and increased photodynamic efficacy *in vitro*. Free Radical Biol. Med..

[12] Usuda J., Kato H., Okunaka T., Furukawa K., Tsutsui H., Yamada K., Suga Y., Honda H., Nagatsuka Y., Ohira T. (2006). Photodynamic Therapy (PDT) for Lung Cancers. J. Thorac. Oncol..

[13] Karim S.P., Adelman R.A. (2013). Profile of verteporfin and its potential for the treatment of central serous chorioretinopathy. Clin. Ophthalmol..

[14] Teiten M.H., Bezdetnaya L., Morliere P., Santus R., Guillemin F. (2003). Endoplasmic reticulum and Golgi apparatus are the preferential sites of Foscan localisation in cultured tumour cells. Brit. J. Cancer.

[15] Wöhrle D., Hirth A., Bogdahn-Rai T., Schnurpfeil G., Shopova M. (1998). Photodynamic therapy of cancer: Second and third generations of photosensitizers. Russ. Chem. Bull..

[16] Arnaut L.G., Pereira M.M., Dabrowski J.M., Silva E.F.F., Schaberle F.A., Abreu A.R., Rocha L.B., Barsan M.M., Urbanska K., Stochel G. (2014). Photodynamic Therapy Efficacy Enhanced by Dynamics: The Role of Charge Transfer and Photostability in the Selection of Photosensitizers. Chem. Eur. J..

[17] Iqbal Z., Masilela N., Nyokong T., Lyubimtsev A., Hanack M., Ziegler T. (2012). Spectral, photophysical and photochemical properties of tetra- and octaglycosylated zinc phthalocyanines. Photochem. Photobiol. Sci..

[18] Nishiyama N., Jang W.-D., Kataoka K. (2007). Supramolecular nanocarriers integrated with dendrimers encapsulating photosensitizers for effective photodynamic therapy and photochemical gene delivery. New J. Chem..

[19] Zorlu Y., Dumoulin F., Bouchu D., Ahsen V., Lafont D. (2010). Monoglycoconjugated water-soluble phthalocyanines. Design and synthesis of potential selectively targeting PDT photosensitisers. Tetrahedron Lett..

[20] Warburg O. (1956). On the Origin of Cancer Cells. Science.

[21] Berthold H.J., Franke S., Thiem J., Schotten T. (2010). Ex Post Glycoconjugation of Phthalocyanines. J. Org. Chem..

[22] Airley R.E., Mobasheri A. (2007). Hypoxic Regulation of Glucose Transport, Anaerobic Metabolism and Angiogenesis in Cancer: Novel Pathways and Targets for Anticancer Therapeutics. Chemotherapy.

[23] Crucius G., Hanack M., Ziegler T. (2013). Synthesis and characterization of [1,4-bis(α,β-galactopyranos-6-yl)phthalocyaninato]zinc(II). J. Porphyrins Phthalocyanines.

[24] Alvarez-Micó X., Calvete M.J.F., Hanack M., Ziegler T. (2007). A new glycosidation method through nitrite displacement on substituted nitrobenzenes. Carbohydr. Res..

[25] Alvarez-Micó X., Calvete M.J.F., Hanack M., Ziegler T. (2007). Expeditious Synthesis of Glycosylated Phthalocyanines. Synthesis.

[26] Hanack M., Iqbal Z., Lyubimtsev A., Özcesmeci I., Özcesmeci M., Ziegler T. (2009). Synthesis of unusual phthalocyanines and naphthalocyanines. J. Porphyrins Phthalocyanines.

[27] Iqbal Z., Hanack M., Ziegler T. (2009). Synthesis of an octasubstituted galactose zinc(II) phthalocyanine. Tetrahedron Lett..

[28] Iqbal Z., Lyubimtsev A., Hanack M., Ziegler T. (2010). Synthesis and characterization of 1,8(11),15(18),22(25)-tetraglycosylated zinc(II) phthalocyanines. J. Porphyrins Phthalocyanines.

[29] Iqbal Z., Lyubimtsev A., Hanack M., Ziegler T. (2009). Anomerically glycosylated zinc(II) naphthalocyanines. Tetrahedron Lett..

[30] Iqbal Z., Lyubimtsev A., Herrmann T., Hanack M., Ziegler T. (2010). Synthesis of Octaglycosylated Zinc(II) Phthalocyanines. Synthesis.

[31] Lyubimtsev A., Iqbal Z., Crucius G., Syrbu S., Ziegler T., Hanack M. (2012). Synthesis of glycosylated metal phthalocyanines and naphthalocyanines. J. Porphyrins Phthalocyanines.

[32] Lv F., He X., Lu L., Wu L., Liu T. (2012). Synthesis, properties and near-infrared imaging evaluation of glucose conjugated zinc phthalocyanine via Click reaction. J. Porphyrins Phthalocyanines.

[33] Haque M.E., Kikuchi T., Kanemitsu K., Tsuda Y. (1987). Selective Deoxygenation via Regioselective Thioacylation of Non-protected Glycopyranosides by the Dibutyltin Oxide Method. Chem. Pharm. Bull..

[34] Card P.J., Reddy G.S. (1983). Fluorinated carbohydrates. 2. Selective fluorination of gluco- and mannopyranosides. Use of 2-D NMR for structural assignments. J. Org. Chem..

[35] Lyubimtsev A., Iqbal Z., Crucius G., Syrbu S., Taraymovich E.S., Ziegler T., Hanack M. (2011). Aggregation behavior and UV-vis spectra of tetra- and octaglycosylated zinc phthalocyanines. J. Porphyrins Phthalocyanines.

[36] Juricek M., Kouwer P.H.J., Rehak J., Sly J., Rowan A.E. (2009). A Novel Modular Approach to Triazole-Functionalized Phthalocyanines Using Click Chemistry. J. Org. Chem..

[37] Fraser R.R., Savard S. (1986). Le lithio-2 naphtalene carbonitrile-1 et ses produits de substitutions. Can. J. Chem..

[38] Pletnev A.A., Tian Q., Larock R.C. (2002). Carbopalladation of Nitriles: Synthesis of 2,3-Diarylindenones and Polycyclic Aromatic Ketones by the Pd-Catalyzed Annulation of Alkynes and Bicyclic Alkenes by 2-Iodoarenenitriles. J. Org. Chem..

[39] Sonogashira K., Tohda Y., Hagihara N. (1975). A convenient synthesis of acetylenes: Catalytic substitutions of acetylenic hydrogen with bromoalkenes, iodoarenes and bromopyridines. Tetrahedron Lett..

[40] Rostovtsev V.V., Green L.G., Fokin V.V., Sharpless K.B. (2002). A Stepwise Huisgen Cycloaddition Process: Copper(I)-Catalyzed Regioselective "Ligation" of Azides and Terminal Alkynes. Angew. Chem. Int. Ed..

[41] Williams D.B.G., Mbatha G.B. (2011). The synthesis and characterisation of carbohydrate-functionalised porphyrazines. Dyes Pigments.

[42] Kalkan A., Bayir Z.A. (2003). Synthesis and Characterisation of Unsymmetrical Porphyrazines Containing Bis(hydroxyethylthio) Substituents. Monatsh. Chem..

[43] Tomoda H., Saito S., Ogawa S., Shiraishi S. (1980). Synthesis of Phthalocyanines from Phthalonitrile with Organic Strong Bases. Chem. Lett..

[44] Amano S., Takemura N., Ohtsuka M., Ogawa S., Chida N. (1999). Total synthesis of paniculide A from d-glucose. Tetrahedron.

[45] Woodward R.B., Logusch E., Nambiar K.P., Sakan K., Ward D.E., Au-Yeung B.W., Balaram P., Browne L.J., Card P.J., Chen C.H. (1981). Asymmetric total synthesis of erythromcin. 1. Synthesis of an erythronolide A secoacid derivative via asymmetric induction. J. Am. Chem. Soc..

[46] Shih-Yuan Lee A., Hu Y.-J., Chu S.-F. (2001). A simple and highly efficient deprotecting method for methoxymethyl and methoxyethoxymethyl ethers and methoxyethoxymethyl esters. Tetrahedron.

[47] Vakalopoulos A., Hoffmann H.M.R. (2001). Chelation, Activation, and Proximity Effects in the Deprotection of Dithianes with ZnBr2. Applications in the Polyketide Field. Org. Lett..

[48] Fujioka H., Minamitsuji Y., Kubo O., Senami K., Maegawa T. (2011). The reaction of acetal-type protective groups in combination with TMSOTf and 2,2-bipyridyl; mild and chemoselective deprotection and direct conversion to other protective groups. Tetrahedron.

[49] Àlvarez Micó X., Vagin S.I., Subramanian L.R., Ziegler T., Hanack M. (2005). New Unsymmetrical Zinc-Phthalocyanine Conjugated with One Azo-Dye Moiety: Synthesis via Opening the Fused Triazole Ring and Spectral Properties. Eur. J. Org. Chem.

[50] Chambrier I., Cook M.J., Mayes D.A., MacDonald C. (2003). NMR spectroscopic evidence for the self-association of some asymmetrically substituted phthalocyanines in solution. J. Porphyrins Phthalocyanines.

[51] Terekhov D.S., Nolan K.J.M., McArthur C.R., Leznoff C.C. (1996). Synthesis of 2,3,9,10,16,17,23,24-Octaalkynylphthalocyanines and the Effects of Concentration and Temperature on Their 1H-NMR Spectra. J. Org. Chem..

[52] Kobayashi N., Ogata H., Nonaka N., Luk’yanets E.A. (2003). Effect of Peripheral Substitution on the Electronic Absorption and Fluorescence Spectra of Metal-Free and Zinc Phthalocyanines. Chem. Eur. J..

[53] Anderson A.B., Gordon T.L., Kenney M.E. (1985). Electronic and redox properties of stacked-ring silicon phthalocyanines from molecular orbital theory. J. Am. Chem. Soc..

[54] Leznoff C.C., Lever A.B.P. (1989). Phthalocyanines: Properties and Applications.

[55] Zhang X.-F., Xi Q., Zhao J. (2010). Fluorescent and triplet state photoactive J-type phthalocyanine nano assemblies: controlled formation and photosensitizing properties. J. Mater. Chem..

[56] Saka E.T., Göl C., Durmuş M., Kantekin H., Bıyıklıoğlu Z. (2012). Photophysical, photochemical and aggregation behavior of novel peripherally tetra-substituted phthalocyanine derivatives. J. Photochem. Photobiol. A.

[57] Kameyama K., Morisue M., Satake A., Kobuke Y. (2005). Highly Fluorescent Self-Coordinated Phthalocyanine Dimers. Angew. Chem. Int. Ed..

[58] Fennel F., Wolter S., Xie Z., Plötz P.-A., Kühn O., Würthner F., Lochbrunner S. (2013). Biphasic Self-Assembly Pathways and Size-Dependent Photophysical Properties of Perylene Bisimide Dye Aggregates. J. Am. Chem. Soc..

[59] Chen Z., Zhong C., Zhang Z., Li Z., Niu L., Bin Y., Zhang F. (2008). Photoresponsive J-Aggregation Behavior of a Novel Azobenzene−Phthalocyanine Dyad and Its Third-Order Optical Nonlinearity. J. Phys. Chem. B.

[60] Sagitullina G.P., Vorontsova M.A., Garkushenko A.K., Poendaev N.V., Sagitullin R.S. (2010). Nitropyridines: X. Palladium-catalyzed cross-coupling of 2-bromo-5-nitropyridine with terminal acetylenes. Russ. J. Org. Chem..

