# Supplementary material for: Synthesis and Spectroscopic Evaluation of Two Novel Glycosylated Zinc(II)-Phthalocyanines
Source: Molecules. 2015 Oct 9;20(10):18367–86. doi: 10.3390/molecules201018367 (PMC6332196; doi:10.3390/molecules201018367)
Supplement: Supplementary file 1 [file molecules-20-18367-s001.pdf]

Supporting Information

NMR-Spectroscopy:

Compound **3**

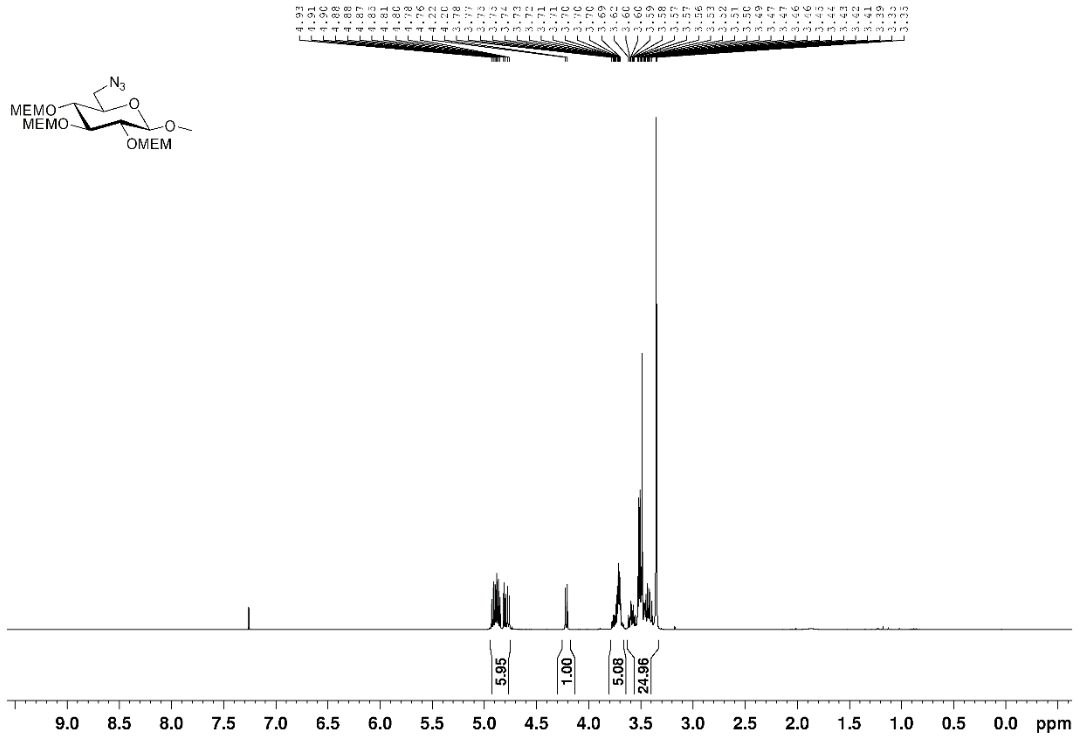

Figure S1. ^{1}H-NMR of compound **3**.

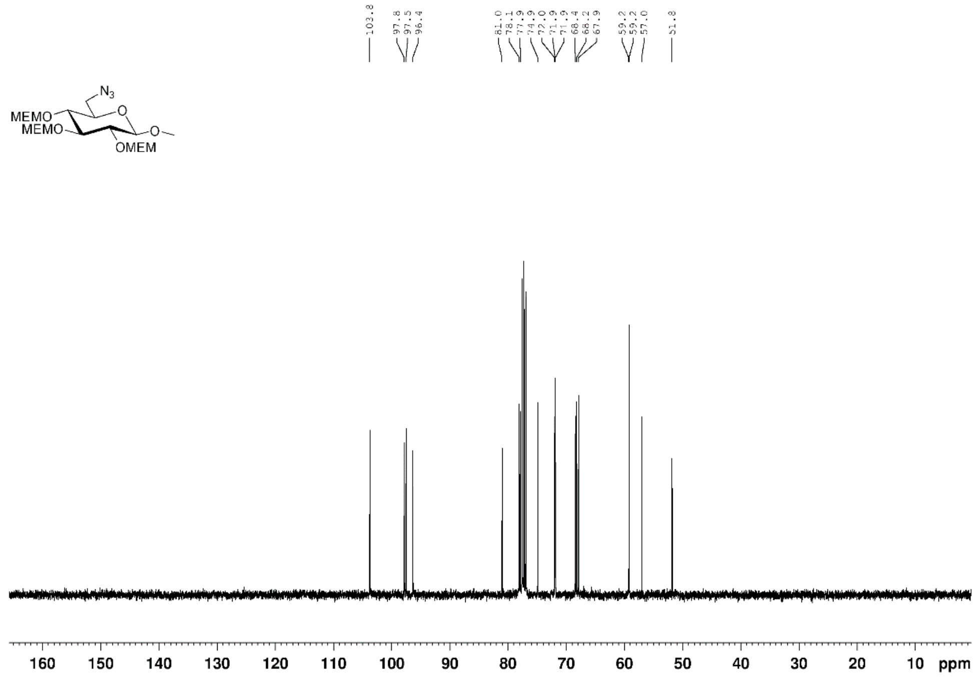

Figure S2. ^{13}C-NMR of compound **3**.

Compound **5a**

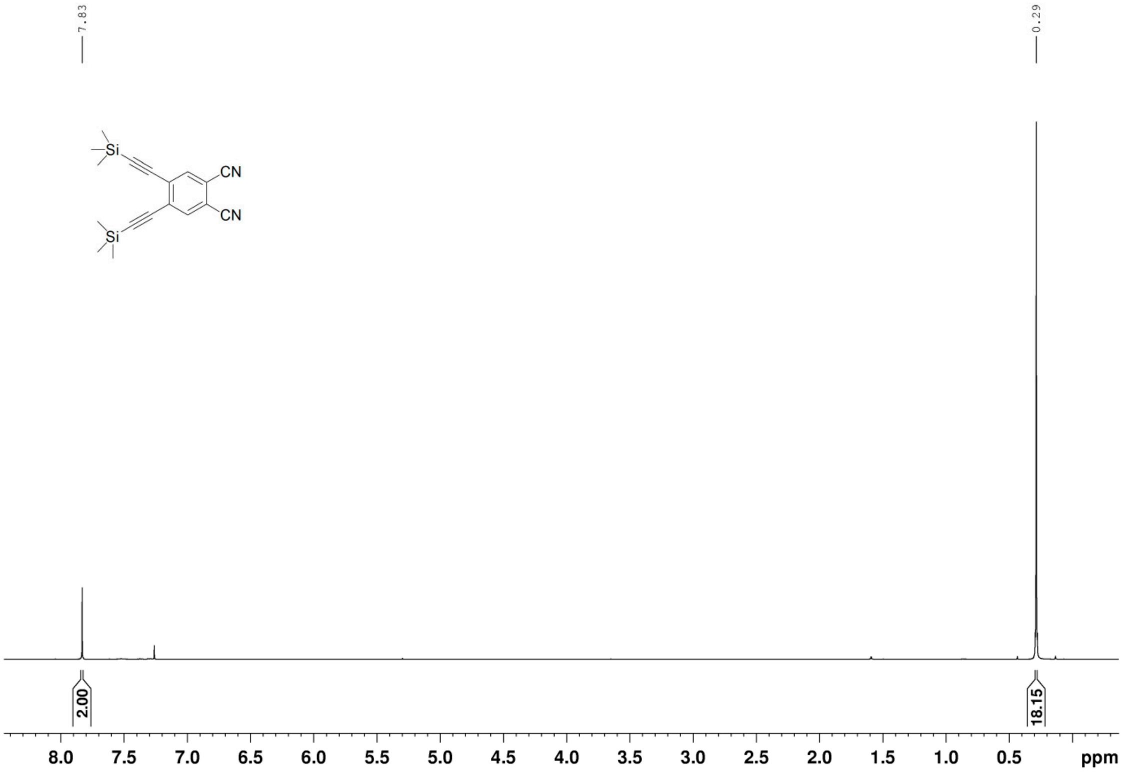

Figure S3. ^{1}H-NMR of compound **5a**.

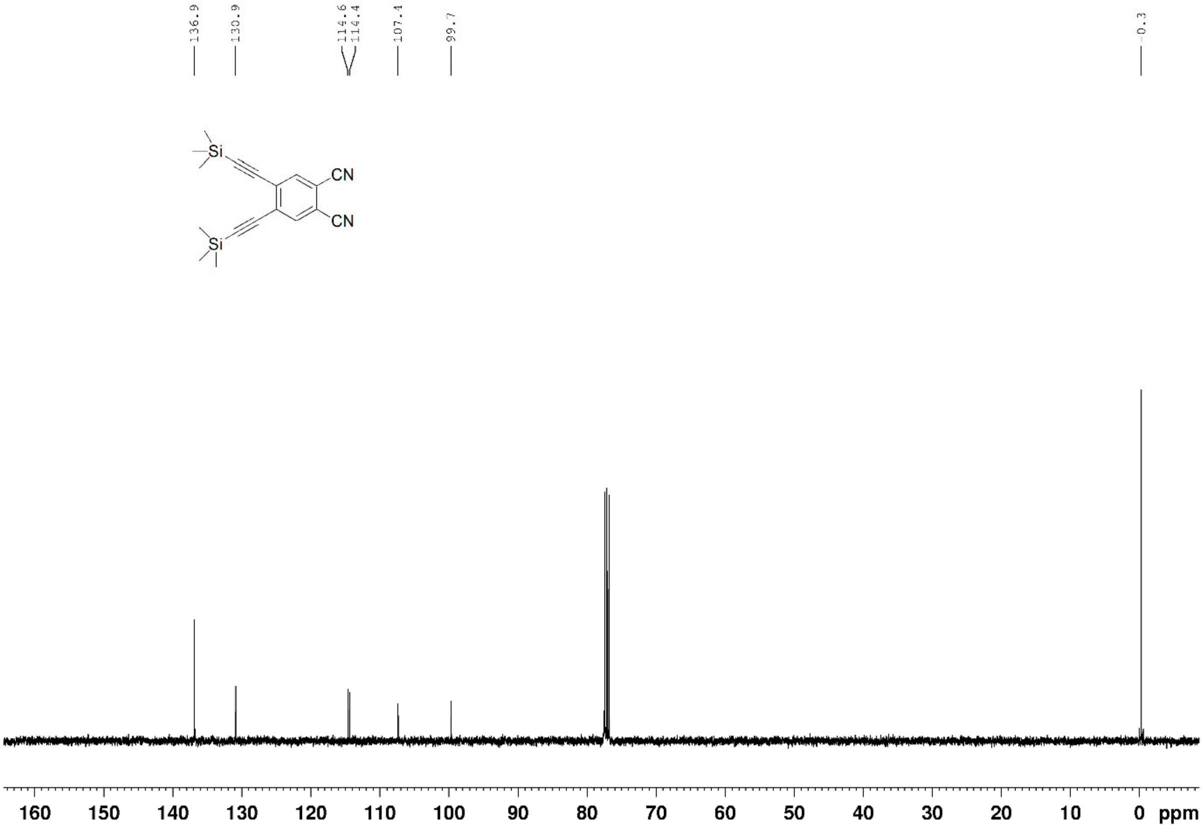

Figure S4. ^{13}C-NMR of compound **5a**.

Compound **5b**

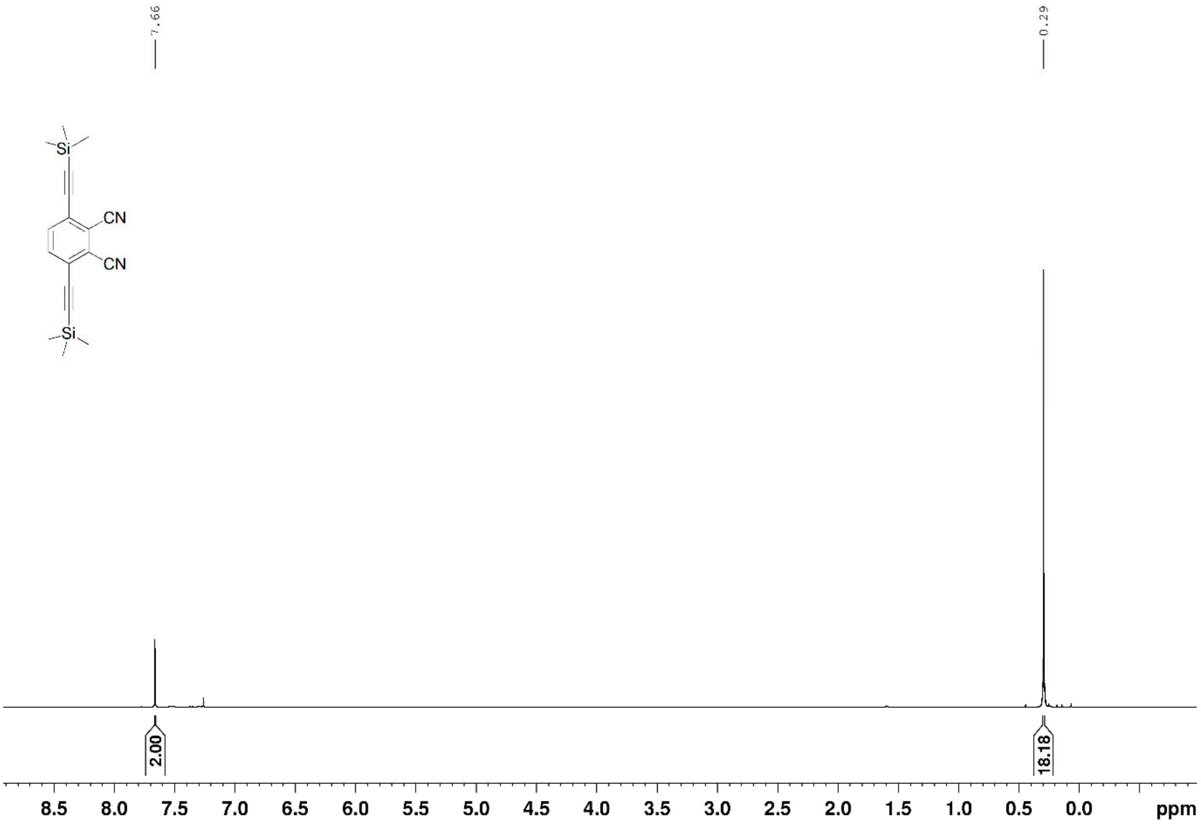

Figure S5. ^{1}H-NMR of compound **5b**.

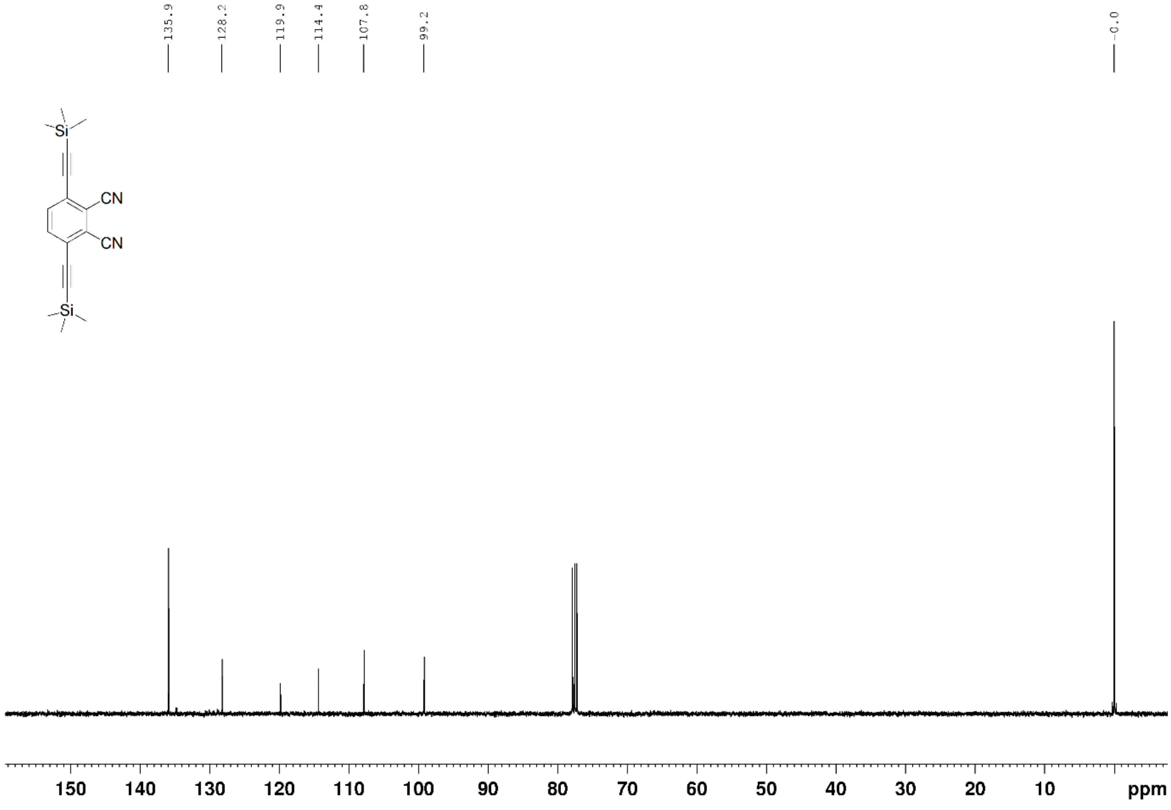

Figure S6. ^{13}C-NMR of compound **5b**.

Compound **6b**

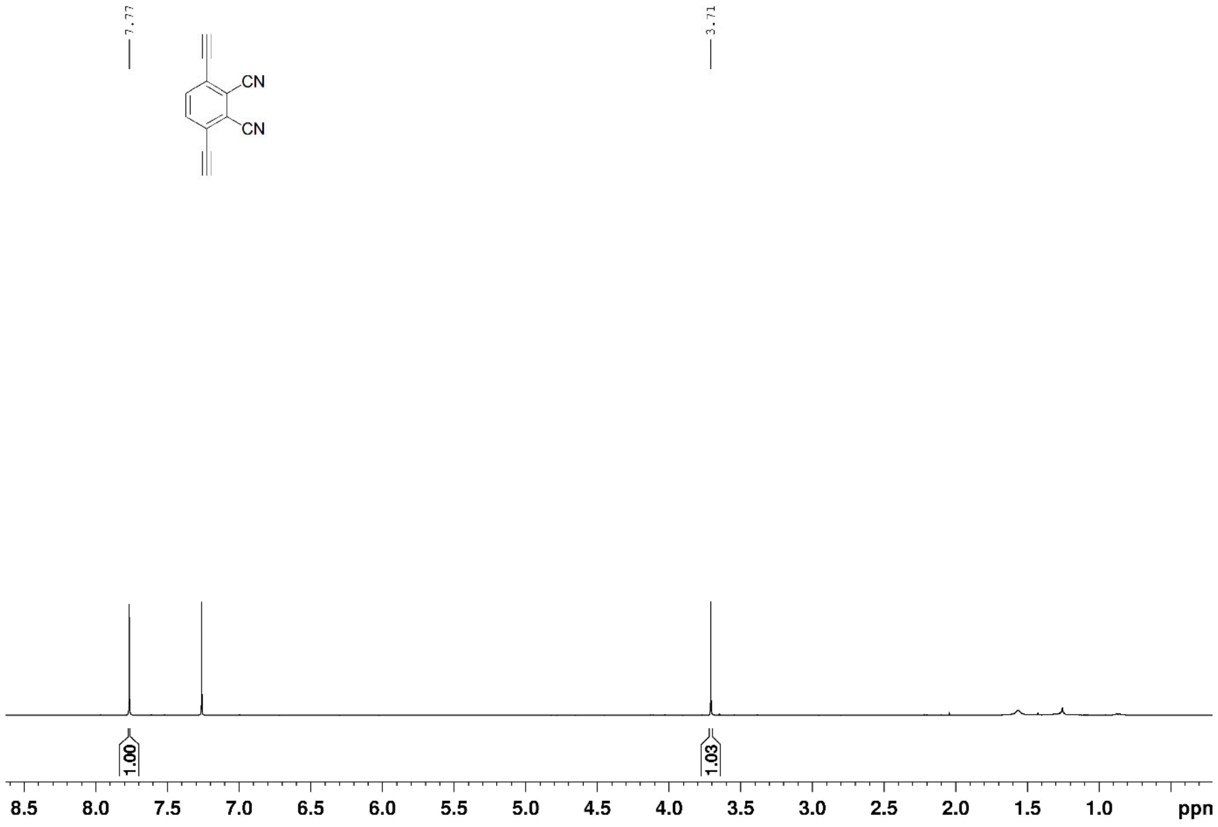

Figure S7. ^{1}H-NMR of compound **6b**.

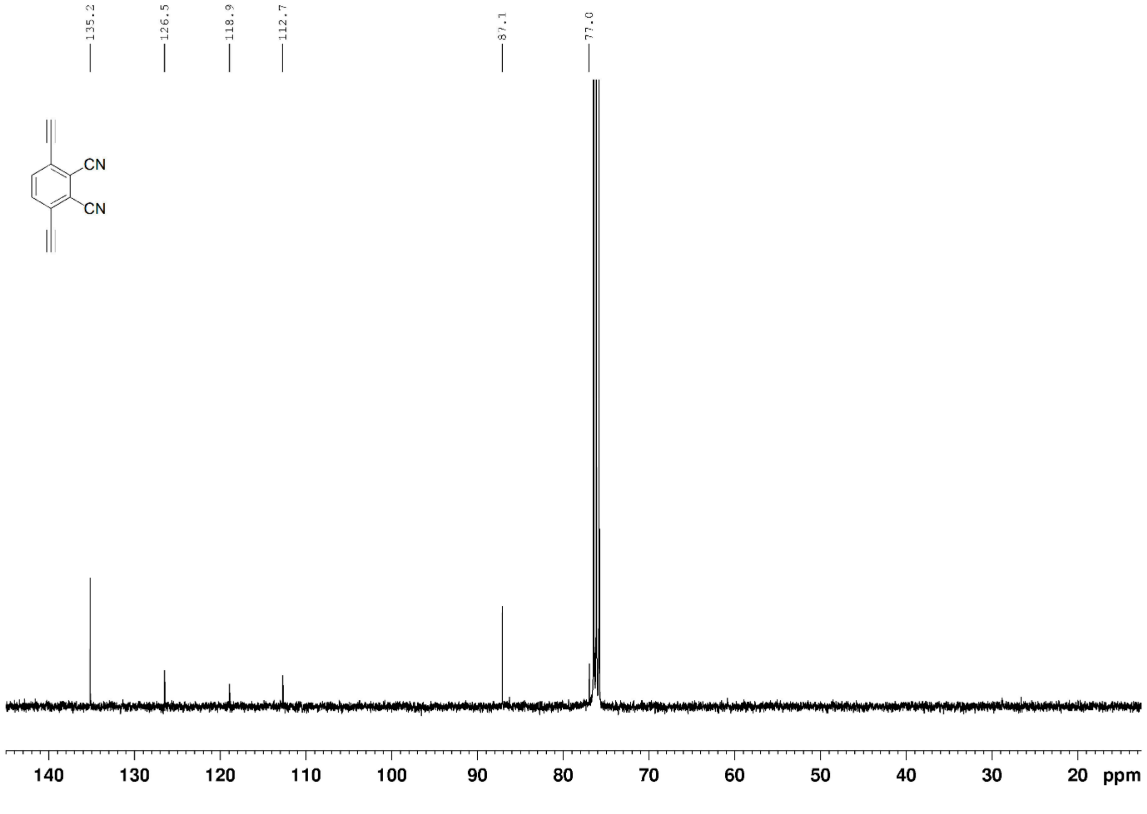

Figure S8. ^{13}C-NMR of compound **6b**.

Compound **7a**

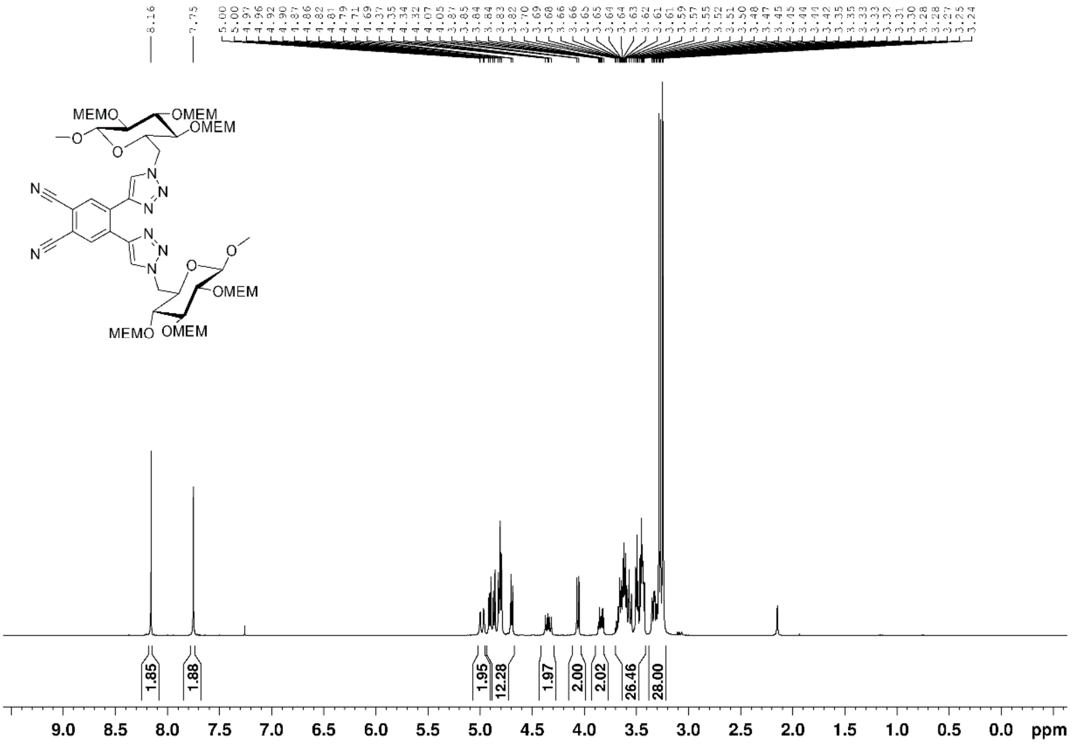

Figure S9. ^{1}H-NMR of compound **7a**.

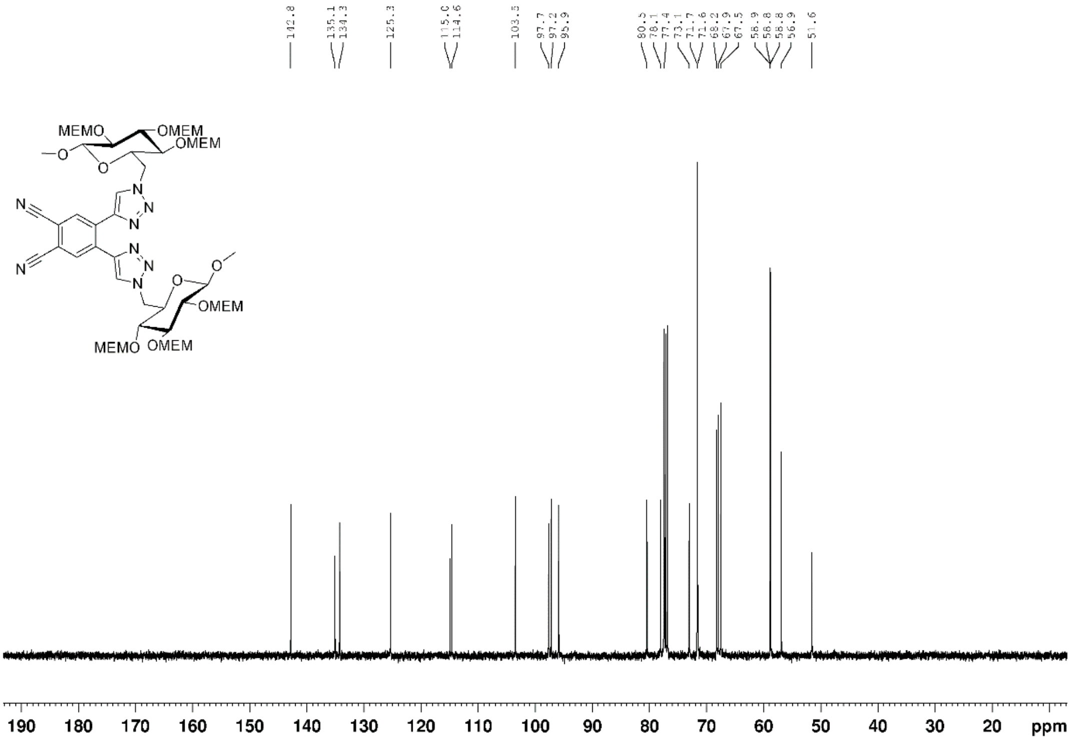

Figure S10. ^{13}C-NMR of compound **7a**.

Compound **7b**

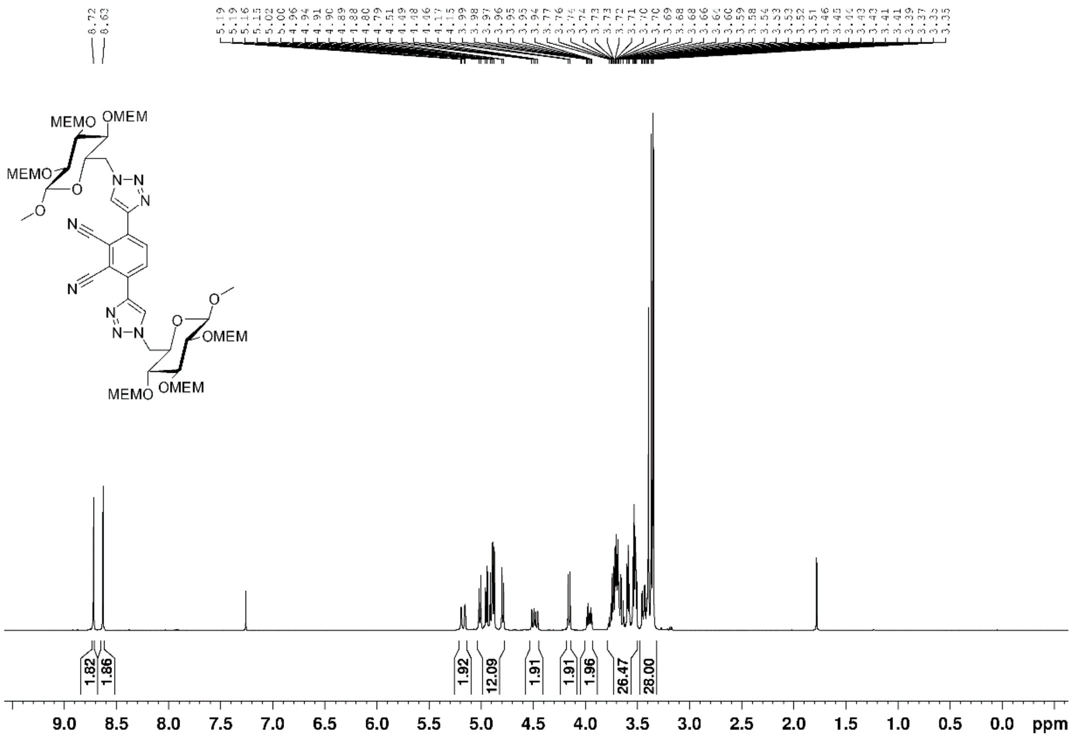

Figure S11. ^{1}H-NMR of compound **7b**.

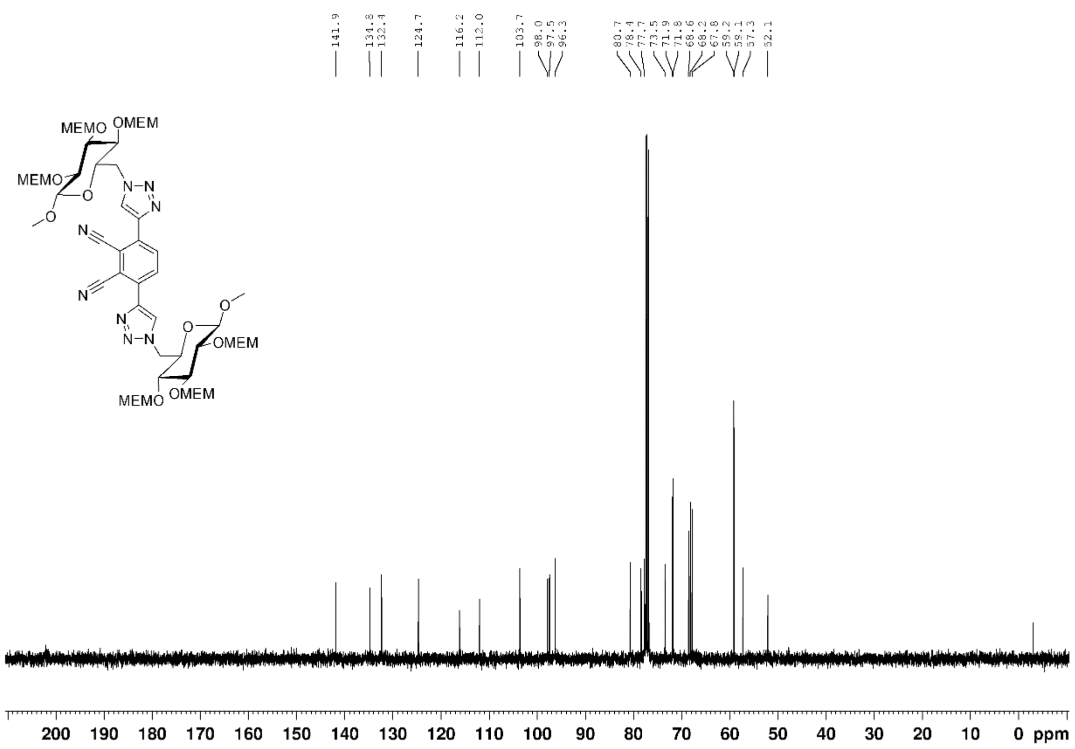

Figure S12. ^{13}C-NMR of compound **7b**.

Compound **8a**

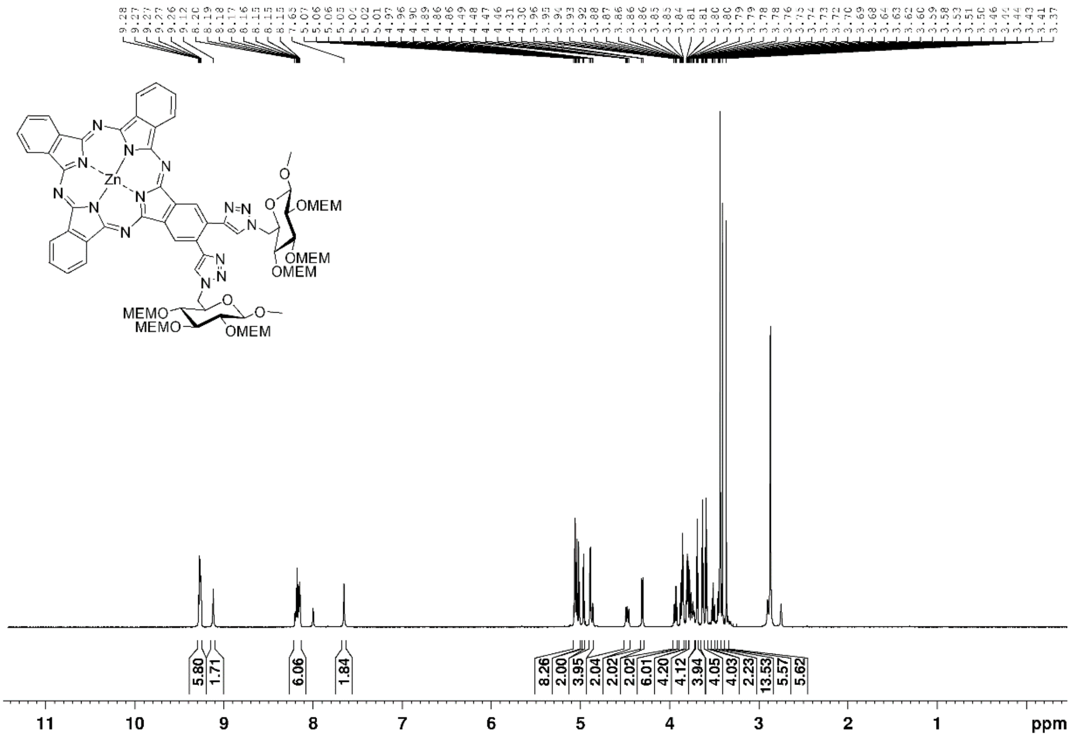

Figure S13. ^{1}H-NMR of compound **8a**.

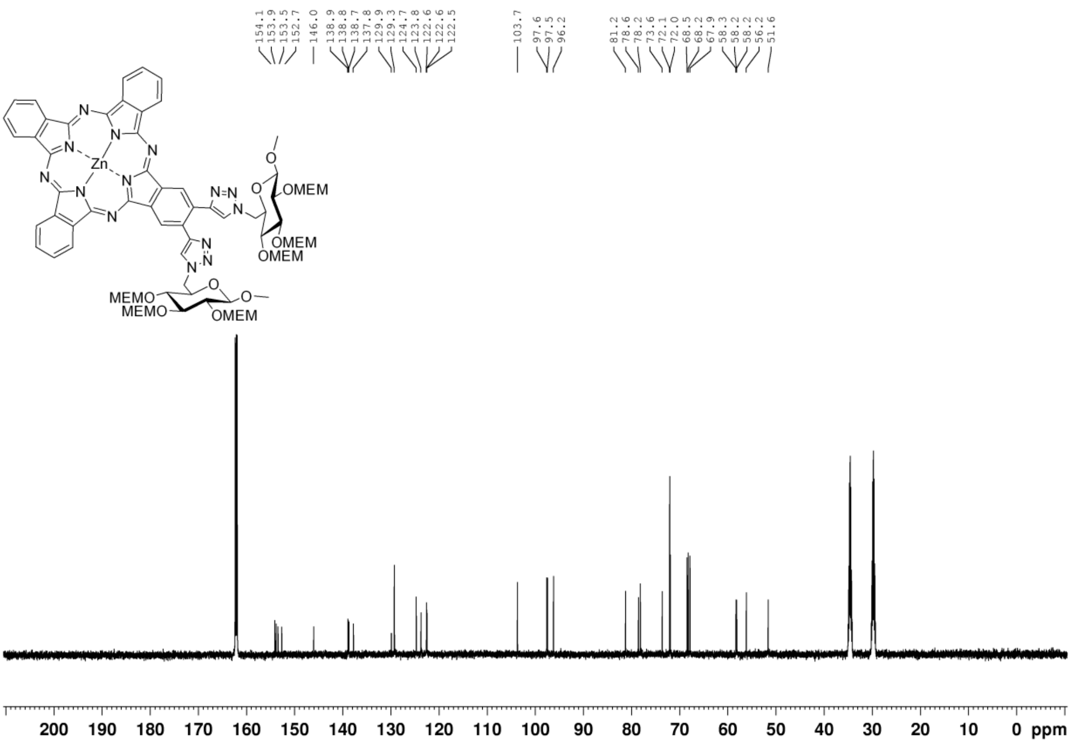

Figure S14. ^{13}C-NMR of compound **8a**.

Compound **8b**

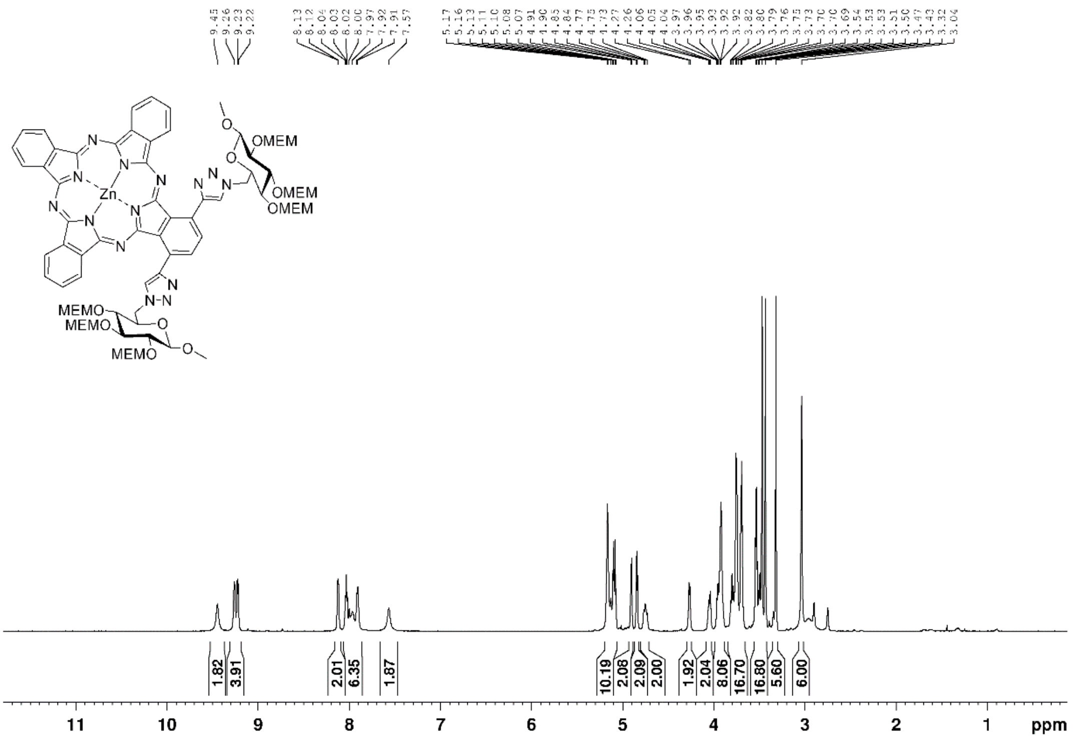

Figure S15. ^{1}H-NMR of compound **8b**.

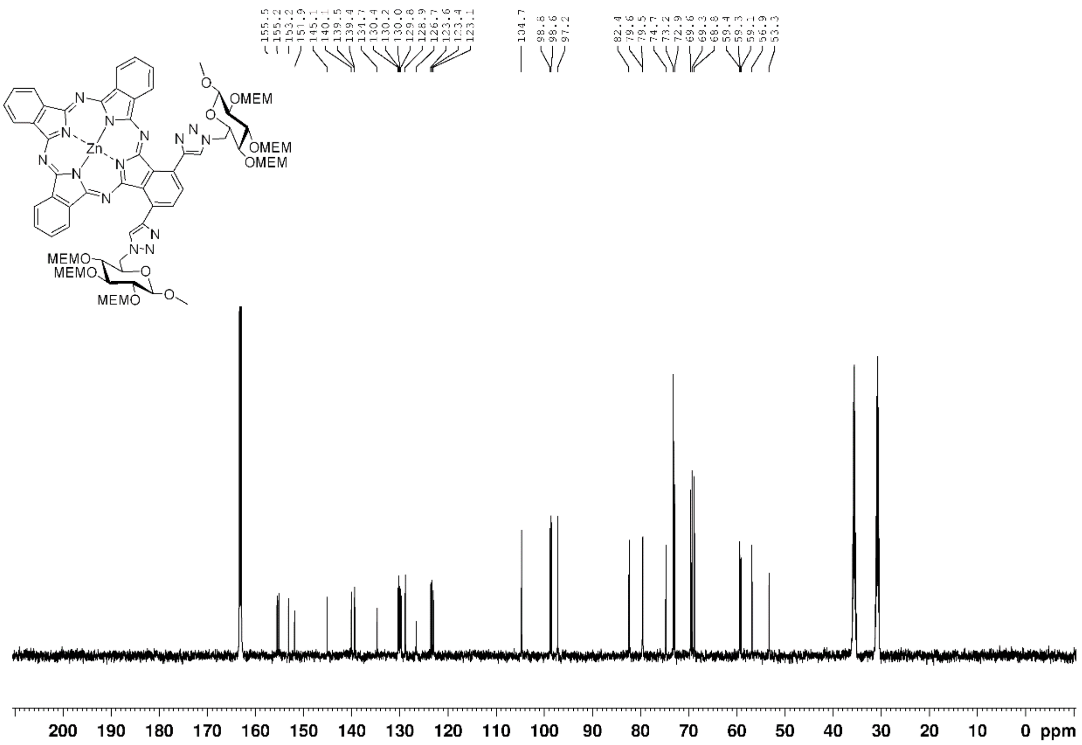

Figure S16. ^{13}C-NMR of compound **8b**.

Compound **9a**

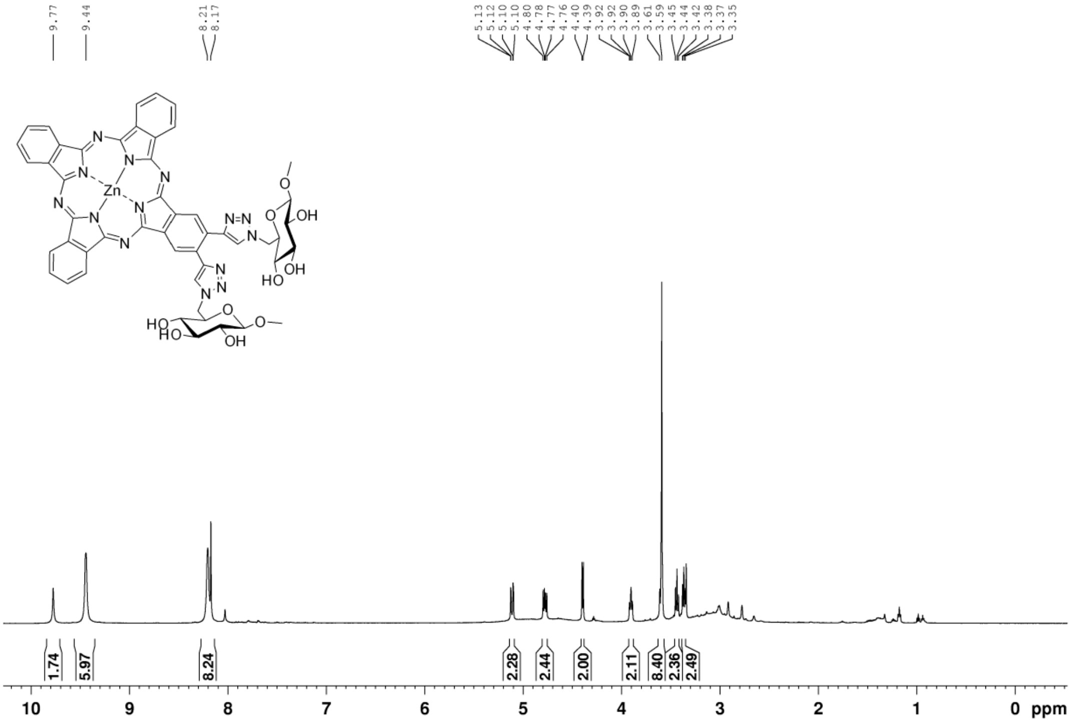

Figure S17. ^{1}H-NMR of compound **9a**.

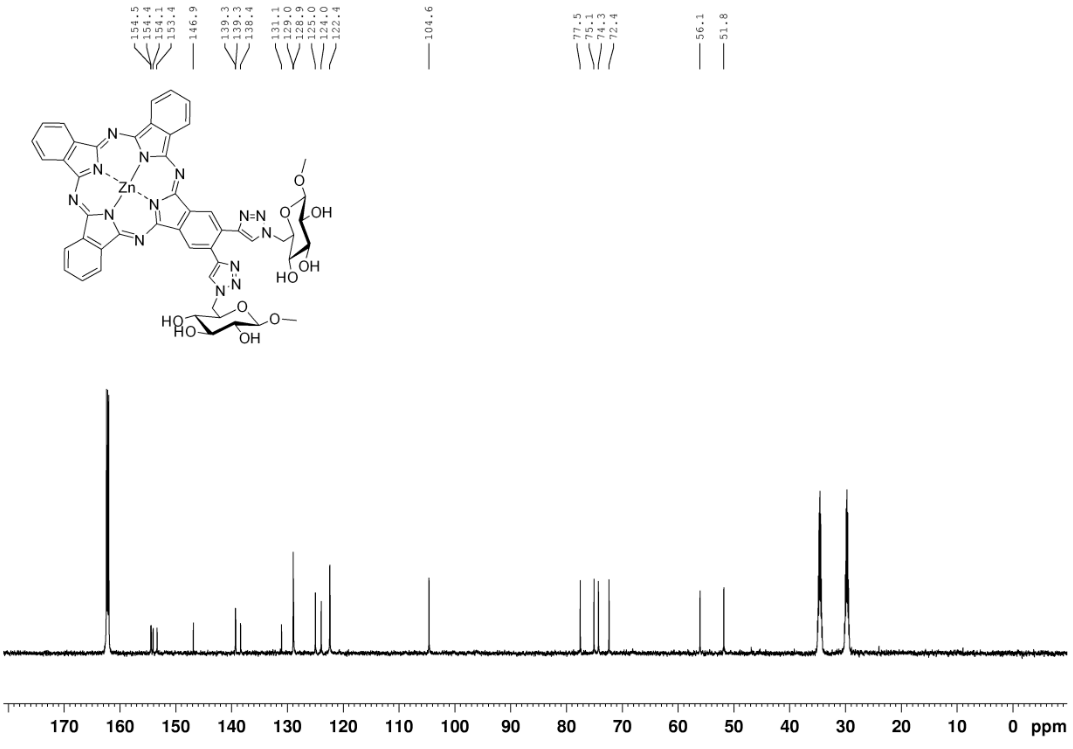

Figure S18. ^{13}C-NMR of compound **9a**.

Compound **9b**

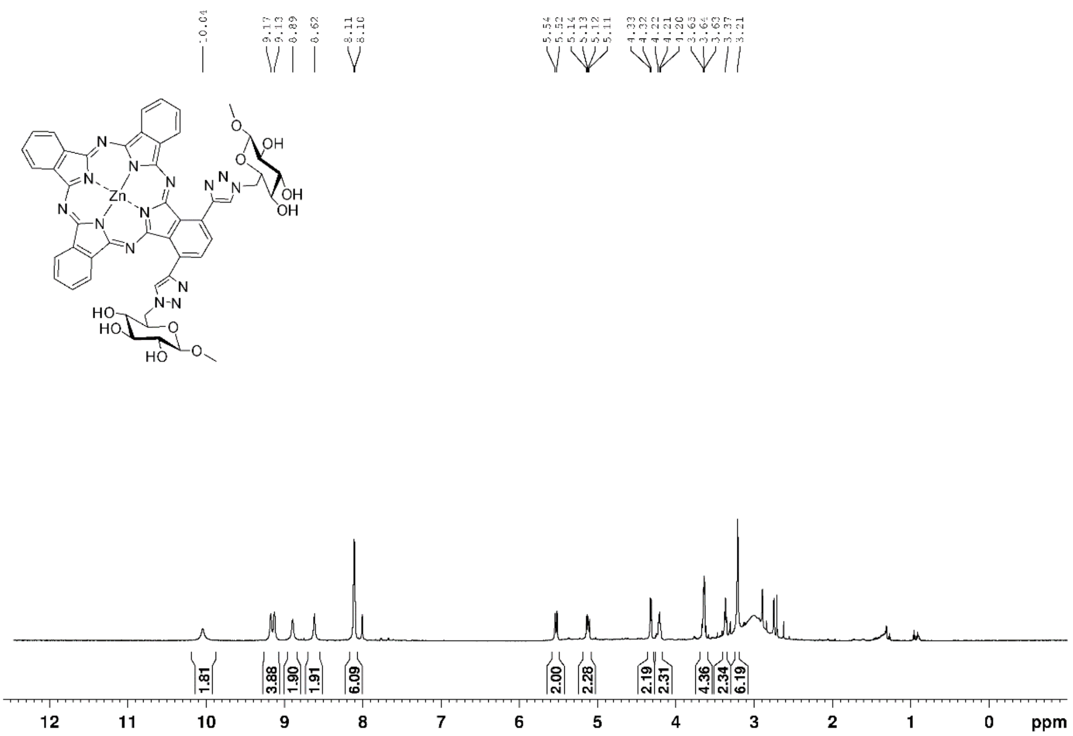

Figure S19. ^{1}H-NMR of compound **9b**.

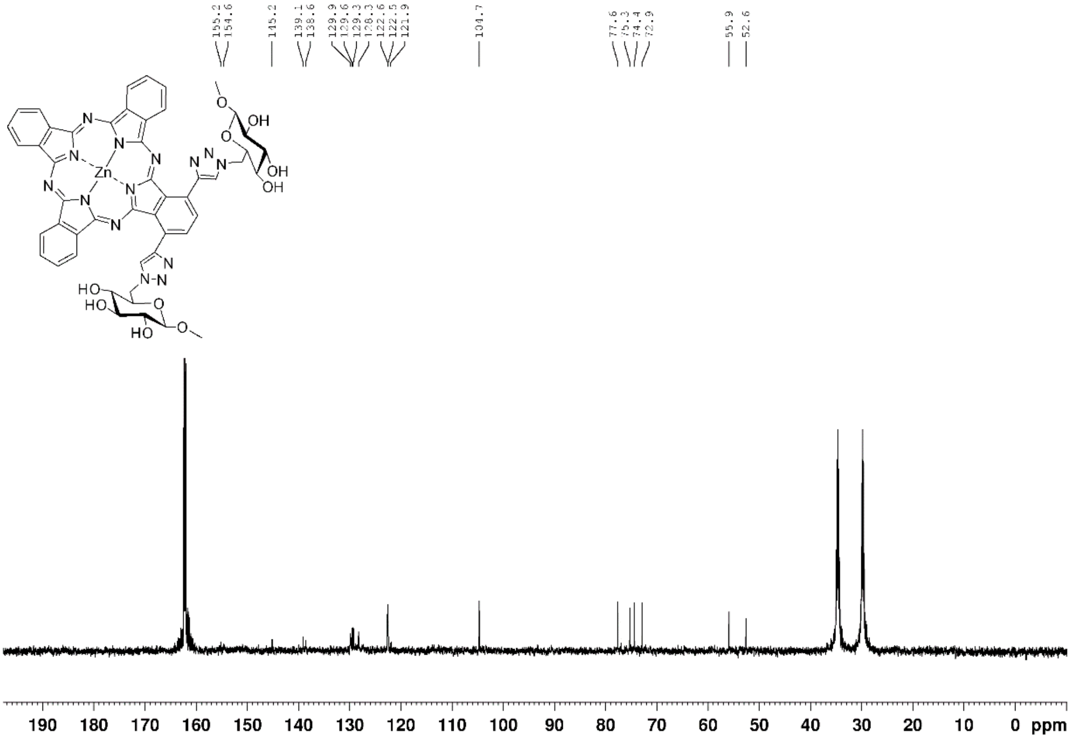

Figure S20. ^{13}C-NMR of compound **9b**.

MALDI-TOF spectra:

Compound **8a**

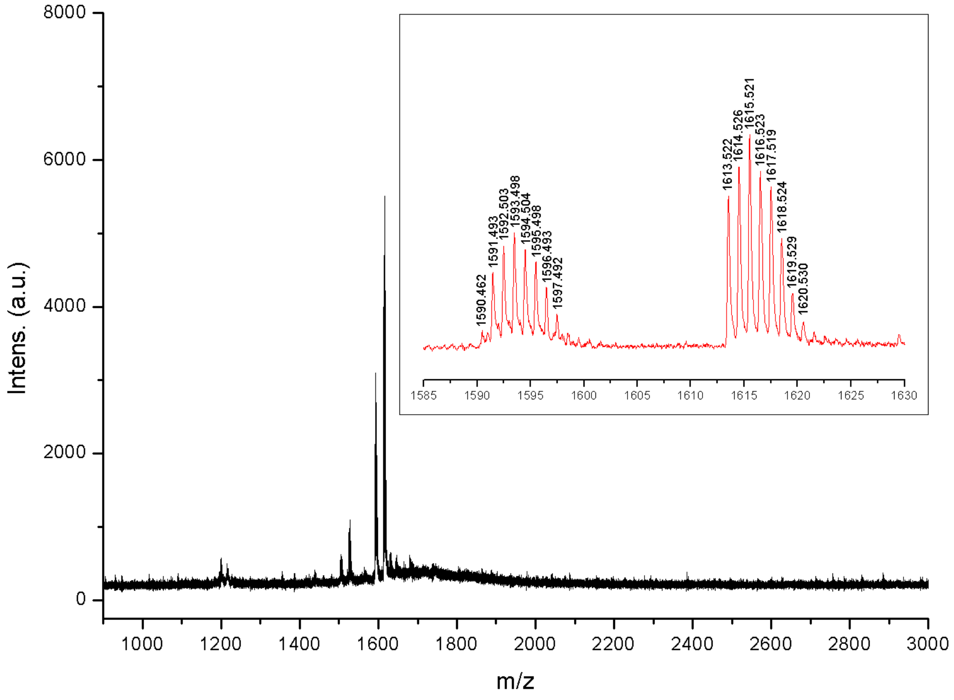

Figure S21. MALDI-TOF spectrum of **8a**.

Compound **8b**

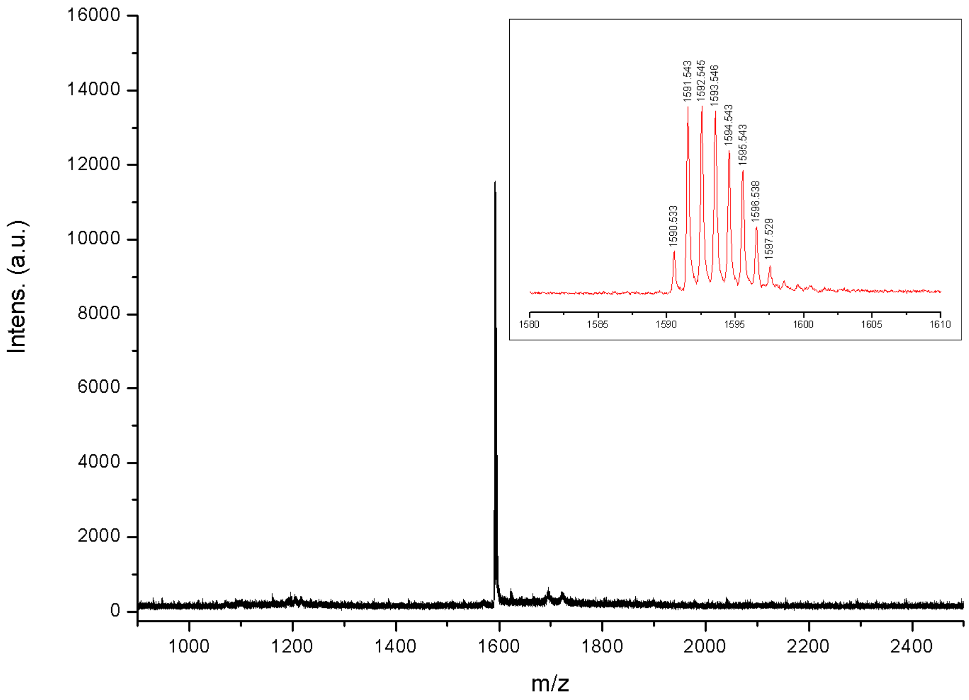

Figure S22. MALDI-TOF spectrum of **8b**.

Compound **9a**

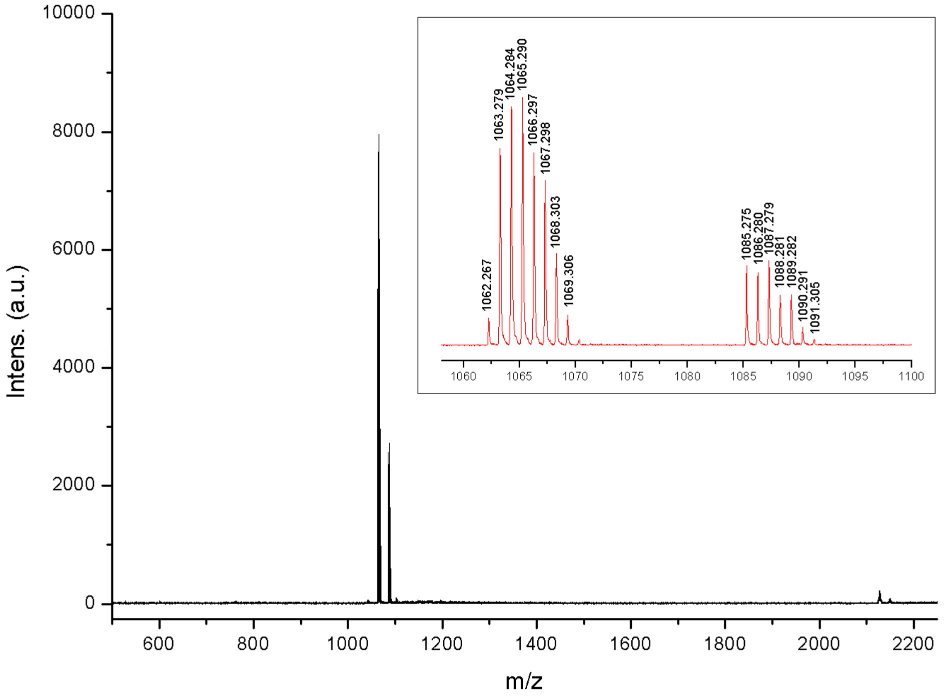

Figure S23. MALDI-TOF spectrum of **9a**.

Compound **9b**

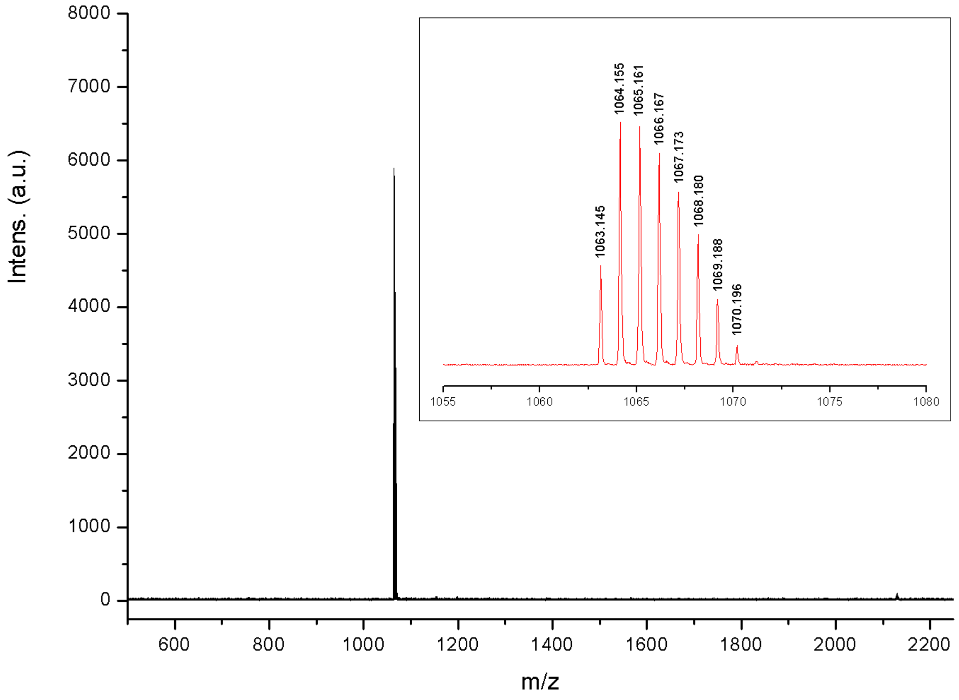

Figure S24. MALDI-TOF spectrum of **9b**.

Fluorescence Lifetime Decay

Compound **8a**

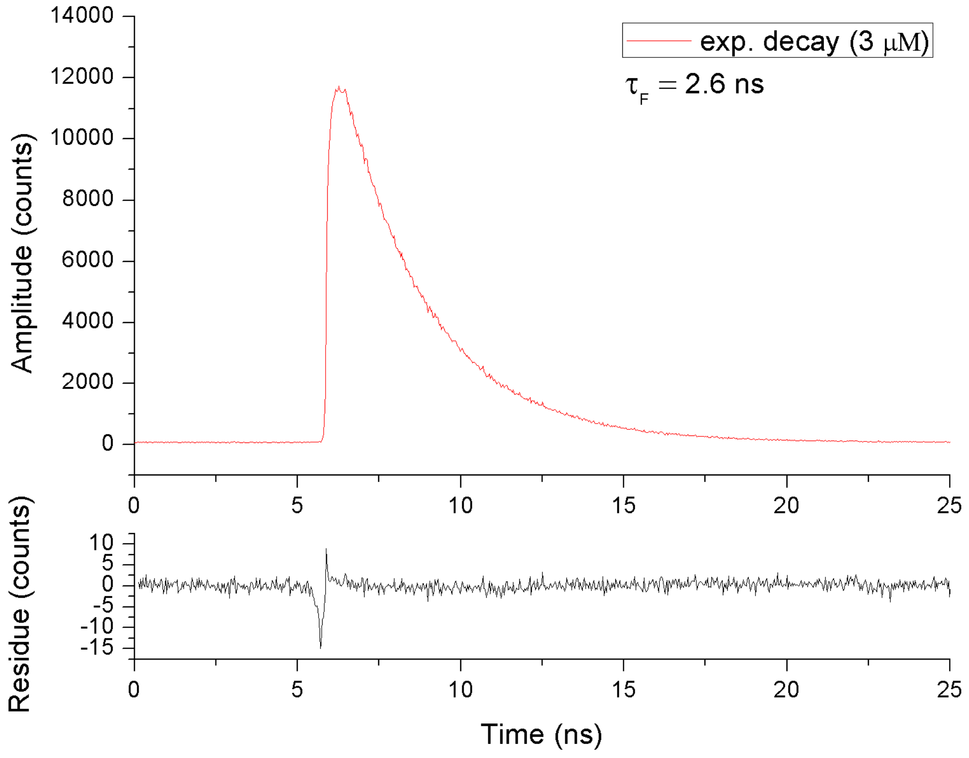

Figure S25. Fluorescence Lifetime Decay of **8a**.

Compound **8b**

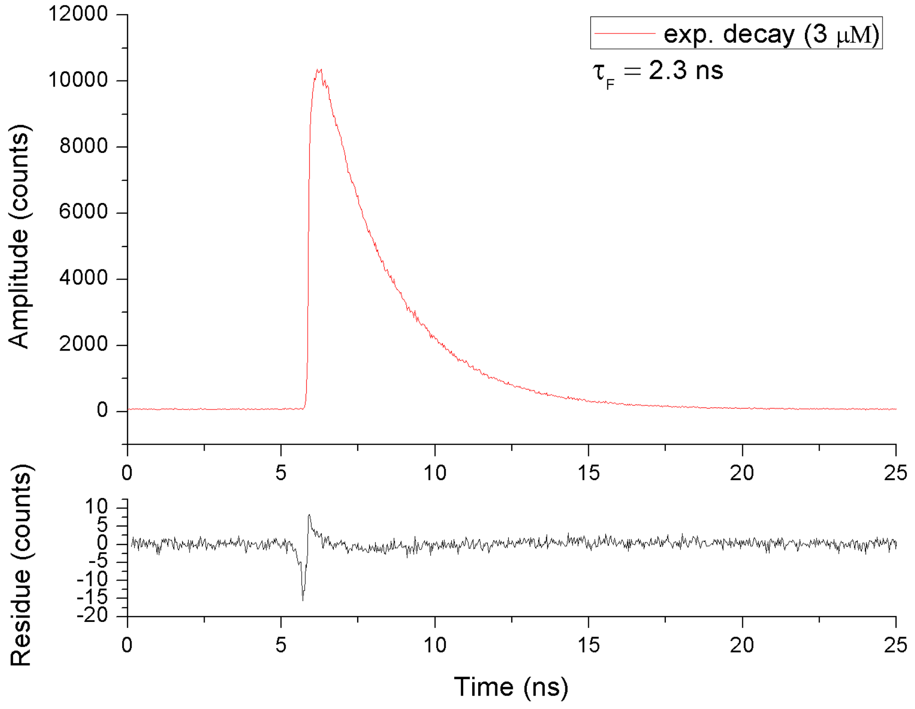

Figure S26. Fluorescence Lifetime Decay of **8b**.

Compound **9a**

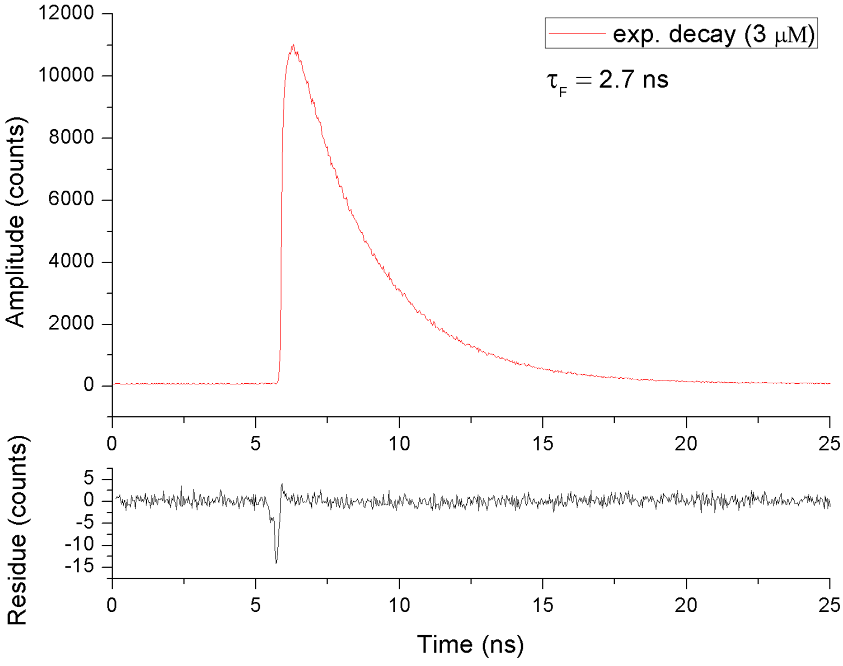

Figure S27. Fluorescence Lifetime Decay of **9a**.

Compound **9b**

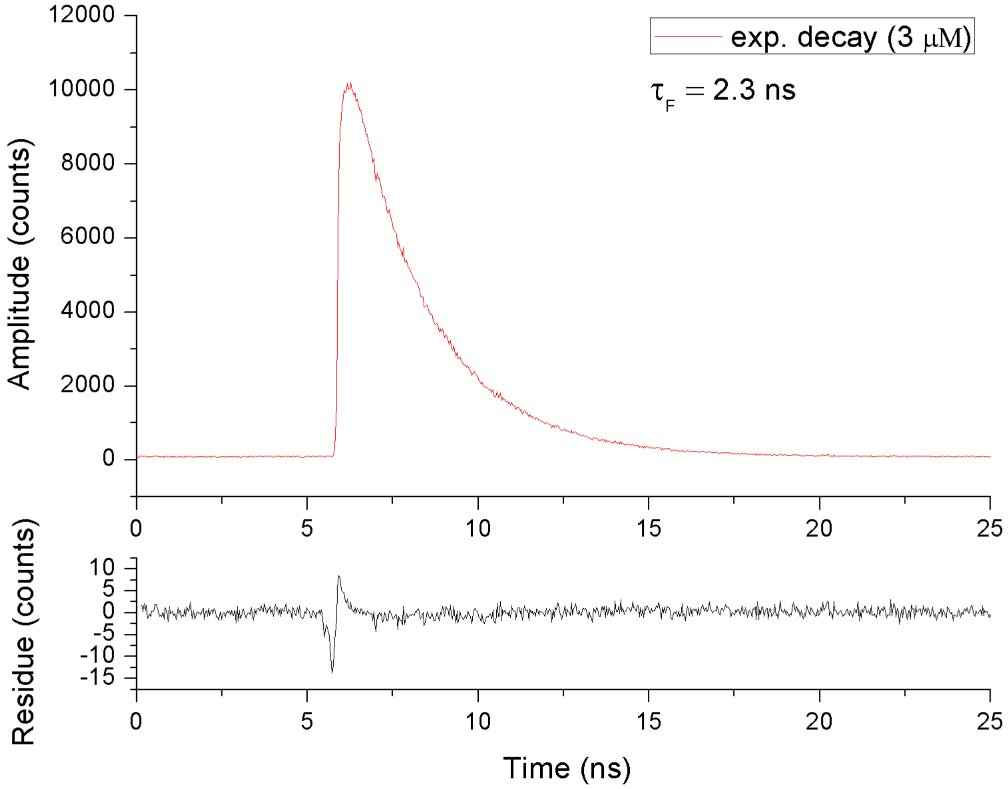

Figure S28. Fluorescence Lifetime Decay of **9b**.

UV-Vis spectra

Compound **8a**

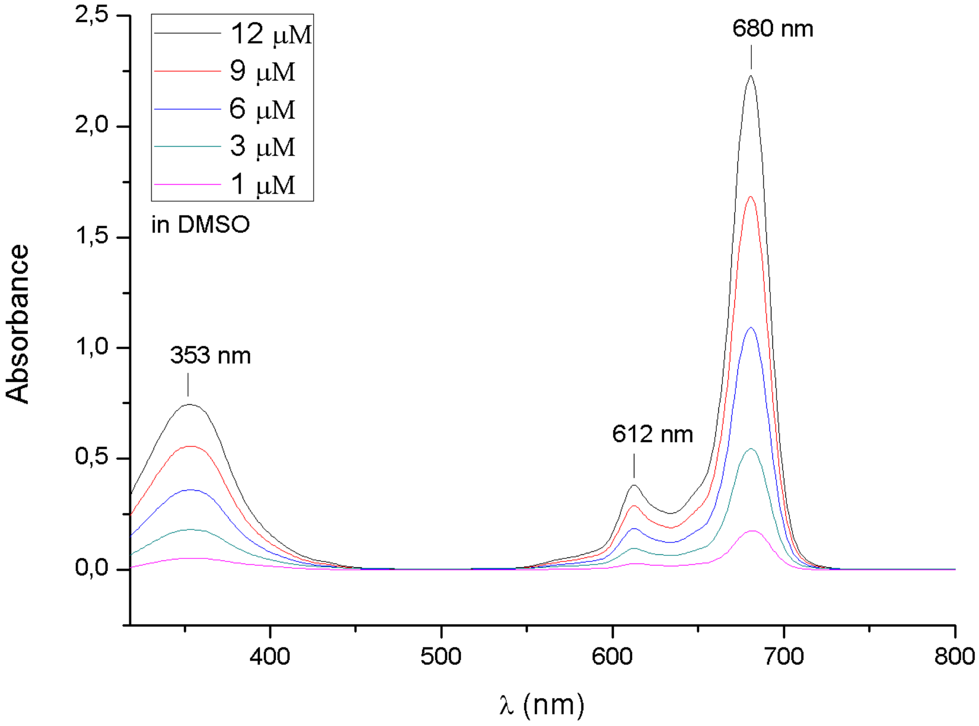

Figure S29. UV-Vis spectrum of **8a**.

Compound **8b**

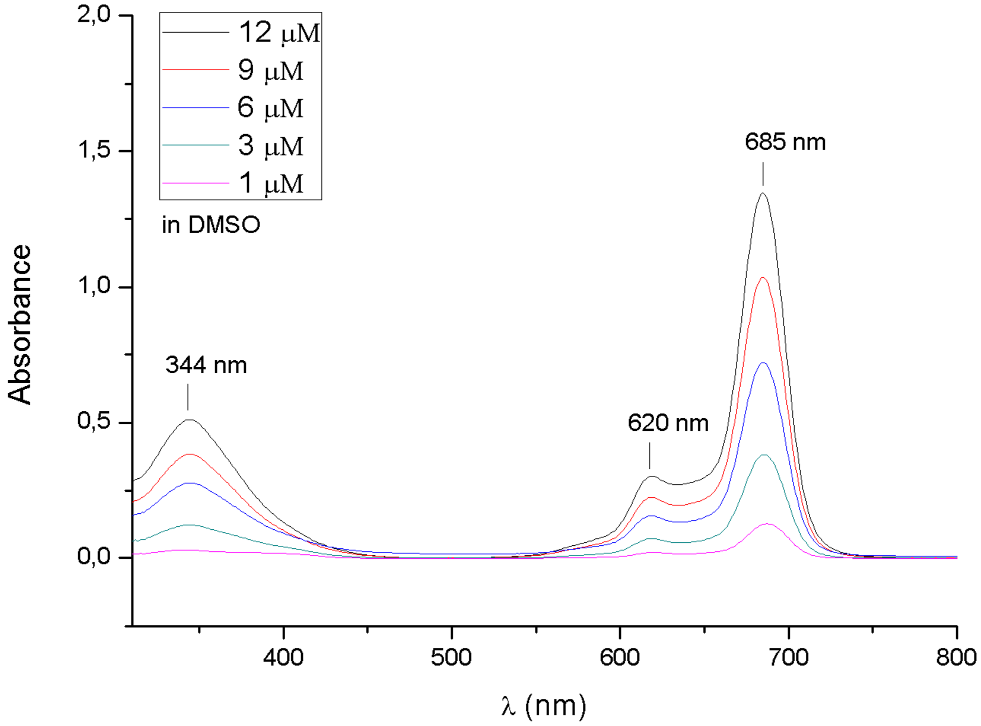

Figure S30. UV-Vis spectrum of **8b**.

Compound **9a**

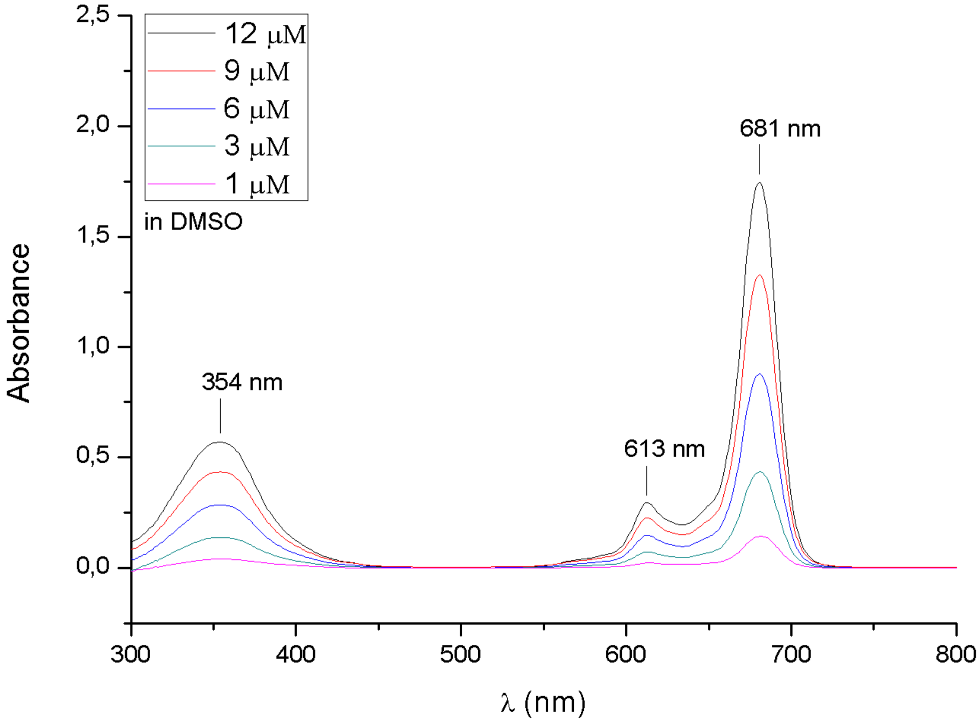

Figure S31. UV-Vis spectrum of **9a** in DMSO.

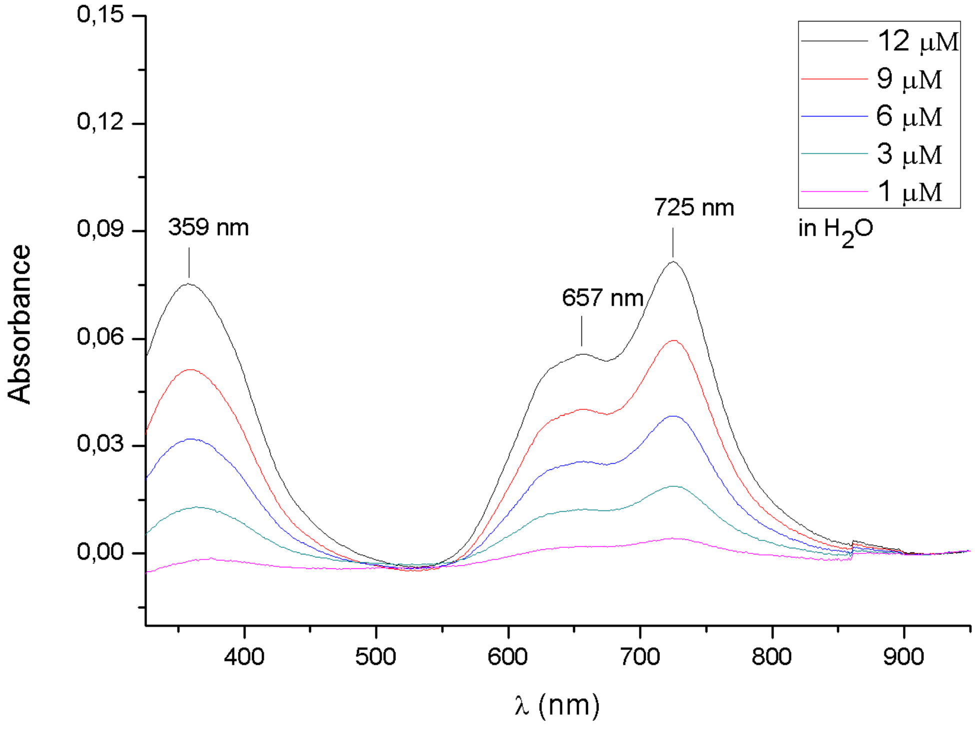

Figure S32. UV-Vis spectrum of **9a** in H_2O.

Compound **9b**

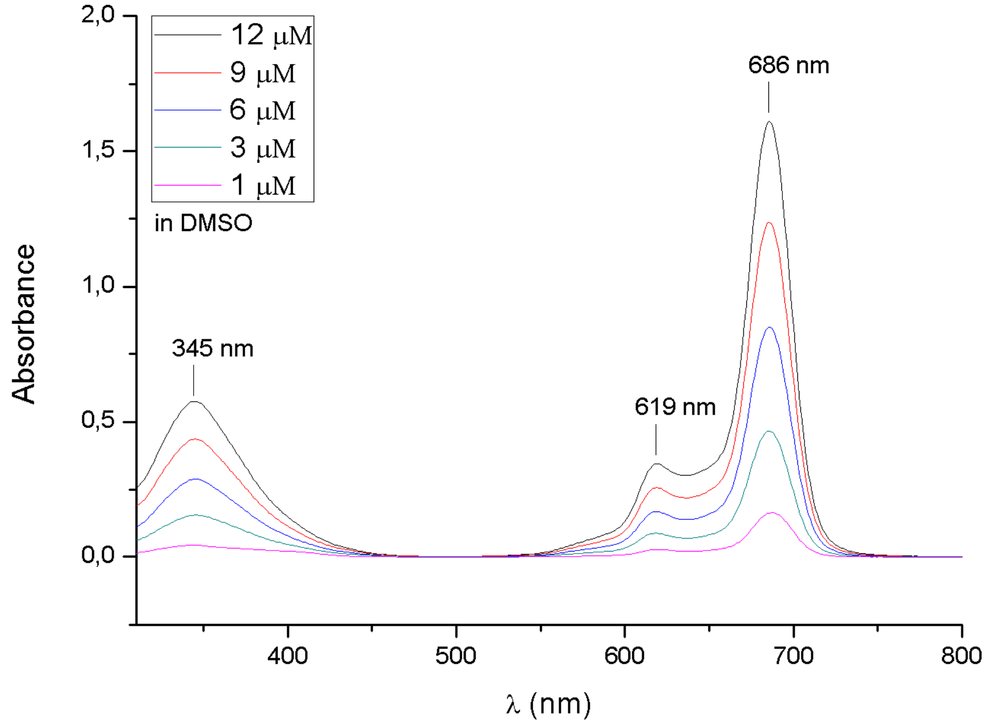

Figure S33. UV-Vis spectrum of **9b** in DMSO

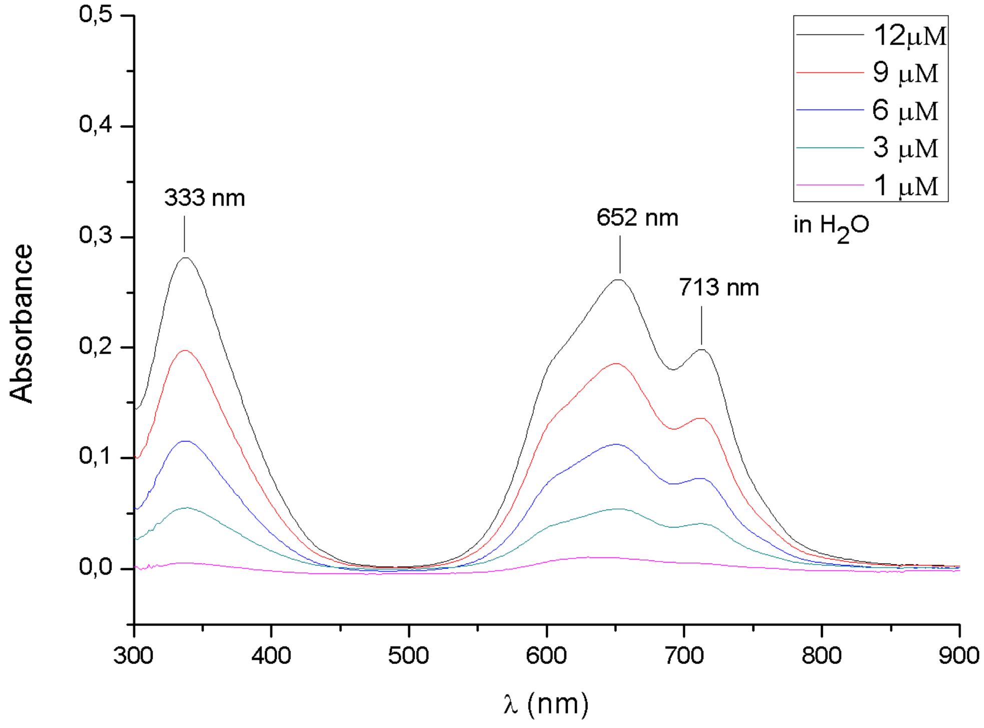

Figure S34. UV-Vis spectrum of **9b** in H_2O.
